# Probing low-energy hyperbolic polaritons in van der Waals crystals with an electron microscope

**DOI:** 10.1038/s41467-017-00056-y

**Published:** 2017-07-21

**Authors:** Alexander A. Govyadinov, Andrea Konečná, Andrey Chuvilin, Saül Vélez, Irene Dolado, Alexey Y. Nikitin, Sergei Lopatin, Fèlix Casanova, Luis E. Hueso, Javier Aizpurua, Rainer Hillenbrand

**Affiliations:** 1 CIC nanoGUNE, 20018 Donostia-San Sebastián, Spain; 2 Materials Physics Center, CSIC-UPV/EHU, 20018 Donostia-San Sebastián, Spain; 3 IKERBASQUE, Basque Foundation for Science, 48013 Bilbao, Spain; 4 Imaging and Characterization Core Lab, King Abdullah University of Science & Technology, 23955 Thuwal, Saudi Arabia; 5 Donostia International Physics Center DIPC, 20018 Donostia-San Sebastián, Spain; 6 CIC nanoGUNE and UPV/EHU, 20018 Donostia-San Sebastián, Spain

## Abstract

Van der Waals materials exhibit intriguing structural, electronic, and photonic properties. Electron energy loss spectroscopy within scanning transmission electron microscopy allows for nanoscale mapping of such properties. However, its detection is typically limited to energy losses in the eV range—too large for probing low-energy excitations such as phonons or mid-infrared plasmons. Here, we adapt a conventional instrument to probe energy loss down to 100 meV, and map phononic states in hexagonal boron nitride, a representative van der Waals material. The boron nitride spectra depend on the flake thickness and on the distance of the electron beam to the flake edges. To explain these observations, we developed a classical response theory that describes the interaction of fast electrons with (anisotropic) van der Waals slabs, revealing that the electron energy loss is dominated by excitation of hyperbolic phonon polaritons, and not of bulk phonons as often reported. Thus, our work is of fundamental importance for interpreting future low-energy loss spectra of van der Waals materials.

## Abstract (web summary)

Here the authors adapt a STEM-EELS system to probe energy loss down to 100 meV, and apply it to map phononic states in hexagonal boron nitride, revealing that the electron loss is dominated by hyperbolic phonon polaritons.

## Introduction

Van der Waals (vdW) materials are a large class of layered materials arranged from individual atomic layers that are bound by (weak) van der Waals interactions^1^. Simple stacking of layers with different physical properties allows for the creation of efficient, compact heterostructures that offer unique possibilities for development of electronic, photonic, and optomechanical devices^1–6^. Much of the vdW materials functionality results from the large anisotropy in the bonding strength of atoms in the direction parallel to atomic layers and across them, and, therefore, is often intimately connected with the corresponding phonons. The investigation of phononic excitations in vdW materials thus requires probing low mid-IR energies at high spatial resolution (few tens of nm and below).

Electron energy loss spectroscopy performed within scanning transmission electron microscopy (STEM-EELS) allows for spatially resolved mapping of a variety of excitations in condensed matter and can provide unprecedented structural information about the sample^7^. However, detecting low-energy excitations (<250 meV) with conventional STEM-EELS is currently more than a challenge. The poor monochromaticity of the (primary) electron beam and the limited resolution of the detection system yield a zero energy loss peak (ZLP) with a typical energy width of about 200 meV and larger^8^. The ZLP acts as a strong background, which prevents measurements of the low-energy loss signals from the sample.

Here, we decreased the ZLP width of a conventional STEM-EELS system to below 50 meV, which allowed us to probe low-energy phononic excitations (down to 100 meV) in vdW materials, particularly in the upper Reststrahlen band^9^ (169–200 meV) of the hexagonal boron nitride (h-BN). We find energy loss peaks that cannot be interpreted as pure transverse (TO) or longitudinal (LO) optical phonon excitations^10^. We, therefore, develop a rigorous classical dielectric response theory to understand the electron energy loss (EEL) in slabs of vdW materials (that show strong optical anisotropy) and demonstrate that it is dominated by the excitation of phonon polaritons, which exhibit a hyperbolic dispersion owing to the layered crystal structure of h-BN. We further show evidence that the electron beam excites hyperbolic surface phonon-polaritons^11, 12^ at the edges of h-BN flakes.

## Results

### STEM-EELS at low-energies

For our experiments, we used an FEI Titan 60–300 STEM-EELS system with 60 keV beam energy, in which we reduced the ZLP width from 200 meV (full width at half maximum, FWHM, according to the tool specifications) to 46 meV. This was achieved essentially by (i) enhancing the monochromaticity of the primary electron beam via the reduction of the anomalous energy broadening due to the Coulomb interaction of the beam electrons (Boersch effect^13^); and (ii) improving the energy resolution of the detection system by a fivefold increase of the spectrometer dispersion (see Methods for details). These modifications suppressed the spectral background (caused by ZLP tails) at energies down to about 100 meV and allowed us to probe excitations in the upper Reststrahlen band of h-BN.

We note that compared to recent developments (using cold field emission) that achieve better resolution^14^, our development is based on a conventional system (using thermionic emission) and offers the advantage of higher and more stable currents^15, 16^, thus faster imaging.

### Spectral map of h-BN flake

**Fig. 1:**
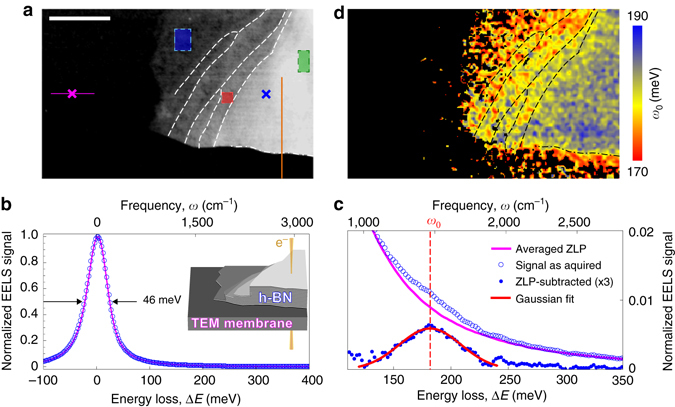
STEM-EELS map of a hexagonal boron nitride flake. **a** STEM HAADF image of the h-BN flake on a TEM membrane. *Brighter areas* correspond to a larger h-BN thickness, *black* is the supporting TEM membrane. Scale bar, 500 nm. *White dashed lines* are guides marking steps on the flake surface, *dash-dotted line* marks the flake edge. *Blue*, *red*, and *green rectangles* mark the area from which the spectra in Fig. 4d were collected. *Orange line* shows where the data for Fig. 6c were taken from. **b** Typical spectra acquired on h-BN (*open circles*) and on an empty TEM membrane (*magenta*); the corresponding locations are marked, respectively, by *magenta* and *blue* crosses in **a**. Each spectrum is normalized to its ZLP maximum. *Inset* shows the 3D sketch of the sample topography from **a**. **c** Close-up view of the same spectrum as in **b** (*open circles*); the *thick magenta* curve shows the ZLP averaged along the *horizontal magenta* line in **a**. *Closed circles* show the spectrum after ZLP subtraction and the *red curve* shows a Gaussian fit. **d** Map of the peak position *ω*_0_. *Black color* marks regions of insufficient signal level (below 2.5x10^−4^ threshold) and of otherwise irregular fits (see Methods for details). *Black dashed* and *dash-dotted lines* are the same eye guides as marked in **a**

The sample studied in this work is an h-BN flake on a 15 nm thick Si_3_N_4_ membrane as shown in Fig. 1a. The black area corresponds to the bare membrane and the bright one to the h-BN flake (see inset for system topology). The brightness in this image is indicative of the h-BN thickness: the dark gray part corresponds to a few nanometers and the white part to about 30 nm, as estimated separately (after STEM-EELS) by atomic force microscopy (AFM) of the same sample (see Methods for details).

Figure 1b shows typical EEL spectra acquired on the bare membrane (magenta) and on the h-BN flake (open symbols). Both spectra are dominated by the ZLP (in which we include the energy loss in the Si_3_N_4_ membrane for simplicity) of nearly identical shape and FWHM of 46 meV, which demonstrates an excellent stability and consistency of our measurements at different spatial locations. A magnified view onto the h-BN spectrum (open symbols in Fig. 1c) reveals an energy loss peak in the range of 120 meV to 250 meV that is not present in the bare membrane spectrum (magenta), and thus indicates an excitation related to phonons in the h-BN. We can isolate this h-BN loss peak (blue dots in Fig. 1c) by subtracting the EEL spectrum of the membrane (see Methods for details). Subsequent Gaussian fitting of the peak (see red curve in Fig. 1c and Methods for details) yields its spectral position *ω*_0_. Isolating and fitting the peak at each pixel yields a spatial map of *ω*_0_, which is shown in Fig. 1d. Intriguingly, we find that the peak position varies throughout the h-BN flake, revealing two notable trends. Firstly, *ω*_0_ depends on the h-BN film thickness gradually blueshifting from ~175 meV at the thinnest part (left flake extremity, see Fig. 1a to correlate with the flake thickness), to about 185 meV at the thickest part near the flake edge (blue zone at the bottom right). Secondly, the h-BN energy loss spectra for aloof beam positions^17^ (i.e., outside the h-BN flake) are redshifted by up to 10 meV compared to the energy loss spectra of a beam passing through the h-BN flake (see bottom right part of the flake in Fig. 1d). Such surprising behavior cannot be explained by the excitation of TO or LO phonons, whose spectral positions are determined solely by the material properties and thus should not depend on the sample thickness or beam position.

### Interaction of fast electrons with vdW materials

**Fig. 2:**
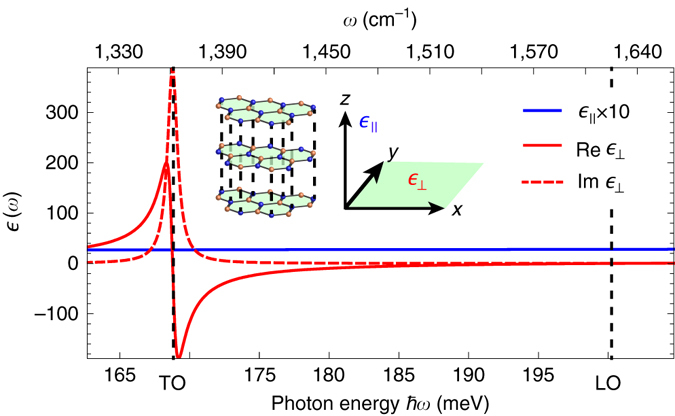
Dielectric permittivity tensor of h-BN in the upper Reststrahlen band. Real (*solid red*) and imaginary (*dashed red*) parts of the principal components of ${\hat{ \epsilon }}$ perpendicular to the h-BN optical axis. For the parallel component, the imaginary part is vanishingly small and only the real part is shown (*blue*). *Inset* illustrates the orientation of the tensor's principal axes with respect to the atomic sheets of h-BN. *Left* and *right vertical dashed lines* mark the TO and LO phonon frequencies, respectively

To understand the variation of the spectral position of the energy loss peak in h-BN, we developed a theory for EEL in thin slabs of vdW materials, based on a classical electrodynamics approach to describe the electron-sample interaction. The electron is represented as a line current (non-recoil approximation), which induces an electromagnetic field, **E**_ind_, in the sample that acts back on the electron^18–20^. **E**_ind_ depends on the dielectric properties of the slab, which are described by the macroscopic permittivity *ϵ*. Our approach rigorously accounts for the strong uniaxial anisotropy of h-BN by treating *ϵ* as a tensor^21, 22^ with two principal components *ϵ*_*||*_ and *ϵ*_⊥_, which describe the dielectric response along and across the optical axis, respectively (the optical axis is perpendicular to the atomic layers of the vdW crystal, see Fig. 2). The EEL probability *Γ* of an electron to lose the energy *ħω* can then be calculated from the work performed by the electron against this induced field along the entire electron trajectory (see Supplementary Note 1). Assuming cylindrical symmetry, it can be written as:

1 $${\it{\Gamma }}\left( \omega \right) = {\int}_{\!\!\!\!\!_0}^{^{{q_c}}} {P\left( {q,\omega } \right){\rm d}q,} $$

where $P\left( {q,\omega } \right){\rm{ = }}\frac{{qe}}{{2{\pi ^2}\hbar \omega }}{\rm R}{\rm e} \left[ {{\int}_{\!\! - \infty }^{^\infty } {{\rm d}{\it{z}}\,{{\hat {\bf z}}}} \cdot {{{\bf E}}_{\rm ind}}\left( {q,z,\omega } \right)\exp \left( {\frac{{ - i\omega z}}{v}} \right)} \right]$ is the probability for the electron to transfer a transverse (to the electron trajectory) momentum *q* upon losing energy *ħω* (*e*, *ħ*, and *v* are the electron charge, the Planck’s constant and the relativistic speed of the electron, respectively). ${q_{\rm c}}\sim 1\,{\dot {\rm A}^{ - 1}}$ is the maximum transverse momentum of electrons that can pass through the collection aperture of the microscope detector. It is determined by a half-aperture collection angle of about 8 mrad of our STEM-EELS instrument. The integral in the expression for *P* is taken over the electron trajectory (parallel to the optical axis ${{\hat {\bf z}}}$ here, see Supplementary Fig. 1) and can be decomposed into three contributions (see Supplementary Note 2):

2 $$P\left( {q,\omega } \right) = {P_{\rm bulk}}\left( {q,\omega } \right) + {P_{\rm guid}}\left( {q,\omega } \right) + {P_{\rm begr}}\left( {q,\omega } \right)$$

**Fig. 3:**
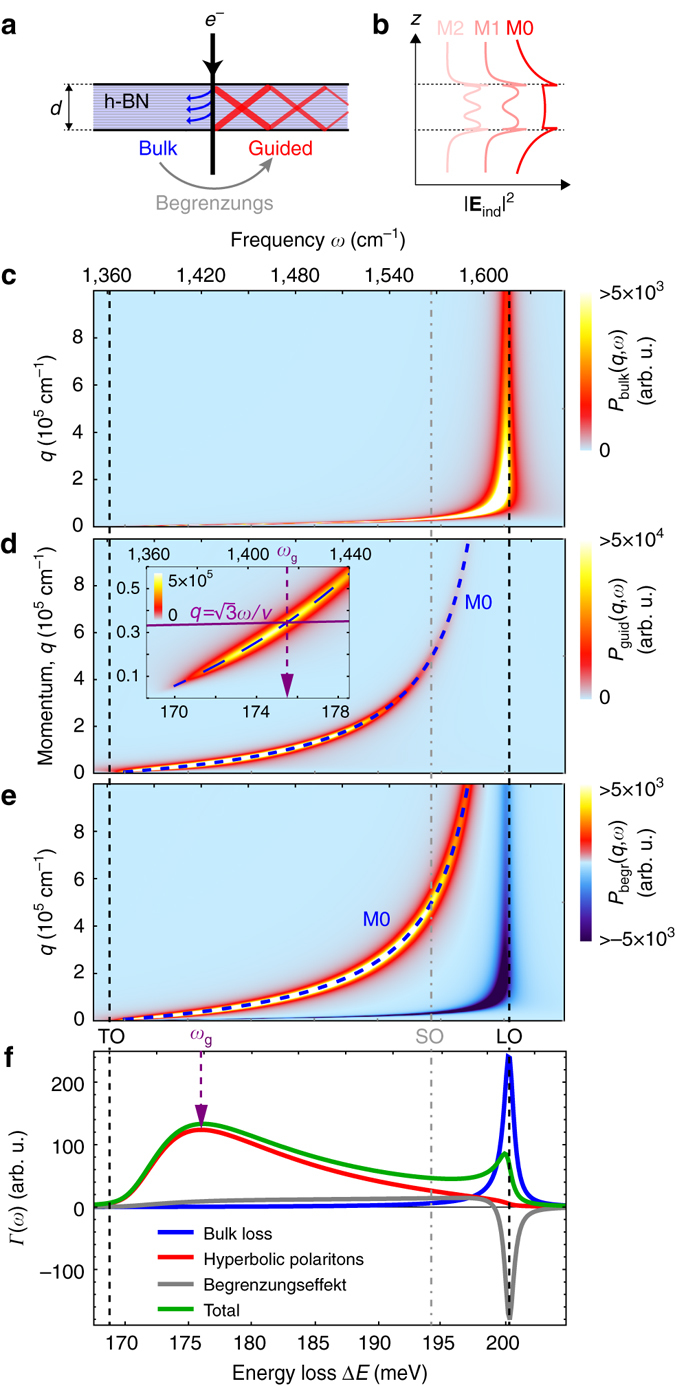
Theoretical EEL probability spectrum of a homogeneous h-BN slab. **a** Schematic representation of the electron energy loss due to the excitation of the bulk phonon (*left*) and guided modes (*right*) in a dielectric slab. **b** Intensity profiles of three guided HPhP modes (M0, M1, and M2). **c** Energy-momentum map *P*_bulk_(*q*,*ω*) calculated for a 30-nm-thick h-BN slab. **d** Same as in **c**, but showing *P*_guid_(*q*,*ω*). **e** Same as in **c**, but showing *P*_begr_(*q*,*ω*). The *dashed curves* in **d** and **e** depict the dispersion of M0 mode of the hyperbolic phonon polariton (calculated according to the Supplementary Eq. (27))^29^. *Inset* in **d** is a close up on *P*_guid_(*q*,*ω*) at low momenta; *dashed purple arrow* shows the energy *ω*_*g*_ at which the line $q = \sqrt 3 \omega /v$ (*solid purple line*, corresponding to the maximum of momentum transfer from electron to the guided mode) intersects with the M0-HPhP mode dispersion. **f** EEL probabilities, *Γ*(*ω*), corresponding to bulk (*blue*), guided-mode (*red*), Begrenzungseffekt (*gray*) losses and their sum (*green*). The *vertical purple arrow* marks the same energy as in the inset of **d**. In all plots, the *vertical dashed lines* mark the TO and LO phonon frequencies; *vertical dot-dashed line* marks the location of the SO energy (corresponds to *ϵ*_⊥_ = −1)

Here, *P*_bulk_ corresponds to the energy loss due to excitations in bulk h-BN (i.e., modes of the unbound medium), similarly to that in isotropic samples^18, 19^, *P*_guid_ is related to the excitation of electromagnetic modes guided along the slab (analogous to the excitation of surface polariton modes in isotropic samples^23, 24^), and *P*_begr_ accounts for the Begrenzungseffekt, i.e., the reduction of the bulk loss due to energy transfer to the guided modes^7, 20, 25^ (see schematics in Fig. 3a).

In Fig. 3c–e we show *P*_bulk_, *P*_guid,_ and *P*_begr_ for the upper Reststrahlen band as a function of *q* and *ω* for a 30-nm-thick h-BN slab, calculated using a nonretarded approximation according to the Supplementary Eqs. (11), (13), and (14) (see Supplementary Note 5 and Supplementary Fig. 5 for retardation effects). *P*_bulk_(*q*, *ω*) depicted in Fig. 3c peaks mainly near the LO phonon frequency, which signifies the excitation of the longitudinal optical phonon (see Supplementary Note 4). *P*_bulk_ thus gives rise to a strong bulk loss peak at the LO phonon frequency in *Γ*_bulk_(*ω*) (blue curve in Fig. 3f).

*P*_guid_ (Fig. 3d) strongly contributes to the energy loss in the lower half of the upper Reststrahlen band, yielding a broad peak in *Γ*_guid_(ω) (red curve in Fig. 3f). *P*_guid_ stems from the excitation of hyperbolic phonon polaritons (HPhPs)^5, 26, 27^ guided along the h-BN slab (see Supplementary Note 3). Hyperbolic polaritons (HPs) are supported in uniaxially anisotropic media when *ϵ*_⊥_ and *ϵ*_*||*_ have opposite signs. Such a strong anisotropy of the dielectric function is typically found in vdW materials^5^. In h-BN it originates from the anisotropic response of optical phonons and gives rise to the lower and upper Reststrahlen bands (Fig. 2). HPs exhibit a dispersion whose isofrequency surfaces are hyperboloids in the three-dimensional momentum space^28^. As a consequence, HPs with momenta much larger than that of a photon can propagate through the volume (in contrast to surface polaritons) of an optically thin slab, resulting in a large number of guided volume modes^27, 29^. Each guided volume mode has a unique profile across the slab (the intensity profiles of the M0 to M2 modes are sketched in Fig. 3b), and is characterized by a unique dispersion that relates its energy to its momentum along the slab, *q*. By plotting the dispersion of the lowest order guided HPhP mode of the h-BN slab (M0-HPhP) in Fig. 3d (blue dashed curve, calculated according to Supplementary Eq. (27)^29^, we observe that it perfectly traces the maximum of *P*_guid_(*q*, *ω*). This finding shows that *P*_guid_(*q*,*ω*) is dominated by the excitation of the M0-HPhP mode. We note that in principle all guided modes are simultaneously excited by the electron beam, with their interference yielding a distinctive zig-zag field pattern (see right hand side of the schematics in Fig. 3a)—a hallmark of HP excitation^26, 30–32^. However, *P*_guid_(*q*,*ω*) exhibits only a single pronounced maximum (Fig. 3d), which lets us conclude that higher order modes are much weaker excited by the electron beam (see discussion below).

*P*_begr_(*q*,*ω*) is depicted in Fig. 3e. This contribution is positive around the dispersion curve of the M0-HPhP mode (dashed blue line in Fig. 3e), but becomes negative at (*q*,*ω*)-values where the bulk loss *P*_bulk_(*q*,*ω*) assumes its maximum values. Consequently, *Γ*_begr_(*ω*) exhibits a strong negative peak at the LO energy (gray curve in Fig. 3f), which in thin slabs is nearly identical in shape and strength to *Γ*_bulk_(*ω*) (blue curve in Fig. 3f).

Importantly, upon summation of all three terms in Eq. (2), the begrenzungseffekt nearly completely cancels the bulk energy loss (as in EEL spectroscopy of isotropic materials^33^, see Supplementary Eq. (30)), leaving only a small shoulder around the LO energy (green curve in Fig. 3f). Therefore, the EEL spectrum of thin vdW slabs is predominantly caused by the excitation of HPs, with the associated spectral peak located neither at TO nor LO frequencies. This central finding of our analysis has been often overlooked, resulting in incorrect interpretation of EEL spectra^14^.

In order to understand the position of the main EEL probability peak associated with the HPhP excitation (marked by the dashed vertical arrow in Fig. 3f), we derive an analytic expression for *P*_guid_ that is valid for optically thin, highly reflective slabs, such as our thin h-BN slab in the upper Reststrahlen band (see Supplementary Note 3):

3 $${P_{\rm guid}}\left( {q,\omega } \right) \approx F\left( {q,\omega } \right)\,{\rm{Im}}\left[ {{q^{ - 1}}{R_{\rm{p}}}\left( {q,\omega } \right)} \right],$$

where $F\left( {q,\omega } \right) = \frac{e}{{{\pi ^2}\hbar v}}\frac{{{q^3}{v^2}}}{{{{\left( {{q^2}{v^2} + {\omega ^2}} \right)}^2}}}$ and *R*_p_(*q*,*ω*) is the reflection coefficient of the slab. The poles of *R*_p_(*q*,*ω*) define the guided modes and the maxima of Im(*R*_p_) trace the modes dispersions in (*q*,*ω*)-space (see Supplementary Fig. 3a)^29^. Thus, Eq. (3) confirms analytically that *P*_guid_ stems from the excitation of guided modes and explains the correspondence between its maxima and the modal dispersions, which we utilized before to identify M0-HPhP as the dominant mode in the energy loss. *F*(*q*,*ω*) in Eq. (3) can be understood as the “probability” for the electron to transfer a momentum *q* to a guided mode upon loosing the energy *ħω* (see Supplementary Note 3). For a given frequency, this probability is the highest when *q* = *q*_max_, with *q*_max_ given by (see Supplementary Fig. 3b)

4 $${q_{{\rm{max}}}} = \sqrt 3 \omega /v.$$

Therefore, the strongest excitation of a polariton mode occurs for the energy *ω*_g_ at which *q*_max_ matches the momentum of the mode (marked by the purple dashed arrow in the inset of Fig. 3d). This energy essentially determines the position of the maximum in *Γ*_guid_(*ω*) (marked by a dashed arrow in Fig. 3f) and can be found geometrically from the intersection of the line defined by Eq. (4) (purple line in the inset) and the guided mode dispersion (M0-HPhP in our case; blue dashed line). This way of finding EEL peak positions is analogous to the concept of momentum matching in optics. However, while in optics one finds the strongest excitation of a guided mode upon matching its momentum with that of the photon, here we match the mode dispersion with *q*_max_. This concept also holds for the excitation of surface polaritons in isotropic materials^19^ (see Supplementary Figs. 2 and 4), making it a valuable tool for analyzing and interpreting the loss peaks in STEM-EELS.

Interestingly, the “probability” function *F*(*q*,*ω*) reaches its maximum at relatively low momenta *q*_max_~3*k*_0_, where *k*_0_ = *ω*/*c* is the photon momentum. For that reason, higher order guided modes that carry momenta much larger than *q*_max_ (see inset in the Supplementary Fig. 3a) are only weakly excited. This explains the observed dominance of the M0-HPhP mode in *P*_guid_ (Fig. 3d) and, correspondingly, the appearance of a single peak in *Γ*_guid_(*ω*) (red curve in Fig. 3f), rather than the appearance of multiple peaks associated with the excitation of several guided modes.

### Thickness dependence

**Fig. 4:**
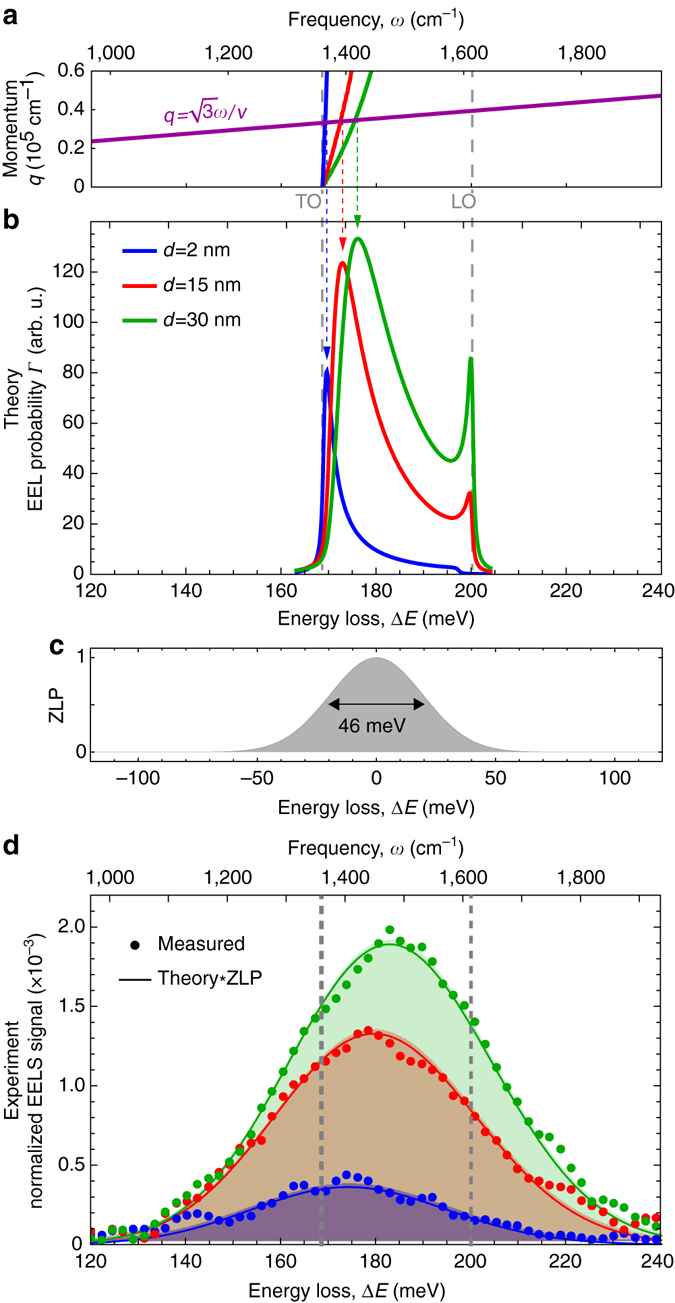
Thickness dependence of h-BN EEL spectra. **a** Dispersions (low energy part) of the fundamental M0-HPhP mode in h-BN films of 2 nm (*blue*), 15 nm (*red*), and 30 nm (*green*) thicknesses. *Blue*, *red*, and *green vertical dashed lines* mark the energies at which these dispersion curves intersect the line $q = \sqrt 3 \omega /v$ (*purple line*), and which determine the positions of spectral maxima of *Γ*_guid_(*ω*). **b** Theoretically calculated EEL probability for the corresponding film thicknesses. **c** A Gaussian with FWHM = 46 meV representing the experimental ZLP. **d** Experimental spectra (*dots*) of h-BN averaged over areas marked by *blue*, *red*, and *green* boxes in Fig. 1a. The *shaded curves* are theoretical EEL spectra obtained by convolving spectra of **b** with the ZLP in **c**. All calculated spectra were scaled by the same factor to correspond with the experiment. In all plots, the *left* and *right vertical dashed lines* mark the TO and LO phonon energies, respectively

Using our theoretical framework, we can explain the thickness dependence of the energy loss spectra of our h-BN sample. To this end, we calculated EEL probability spectra for 2, 15, and 30 nm thick h-BN slabs according to Eq. (1). For all spectra (Fig. 4b) we observe a dominant loss peak due to excitation of the M0-HPhP mode. Most importantly, this peak shifts to lower energies with decreasing slab thickness, which is in good agreement with our experimental observations (Fig. 1d). The peak shift can be explained by the increasing momentum *q* carried by the M0-HPhP mode when decreasing the thickness of the h-BN slab^29, 31^. As can be seen in Fig. 4a, the intersection point of the M0-HPhP mode dispersion (blue, red, green curves) with the line defined by Eq. (4)—and thus the maximum of the loss peak—shifts to lower energies.

For a quantitative comparison between experiment and theory we convolve the calculated spectra of Fig. 4b with a Gaussian of 46 meV FWHM (Fig. 4c), in order to take into account the spectral resolution determined by the experimental ZLP:

5 $${\rm{EEL}} = {\rm{ZLP}} * {\it{\Gamma }}.$$

Figure 4d shows the convolved calculated h-BN EEL spectra (shaded curves) for 2 nm, 15 nm, and 30 nm thick h-BN slabs, respectively. The peak position and strength match remarkably well with the experimental h-BN EEL spectra (dots in Fig. 4d) obtained from the areas with the same h-BN flake thicknesses (marked by blue, red, and green rectangles in Fig. 1a, respectively), thus corroborating our theoretical analysis and explanation of the origin of the peak shifts.

### EEL near edges of vdW materials

**Fig. 5:**
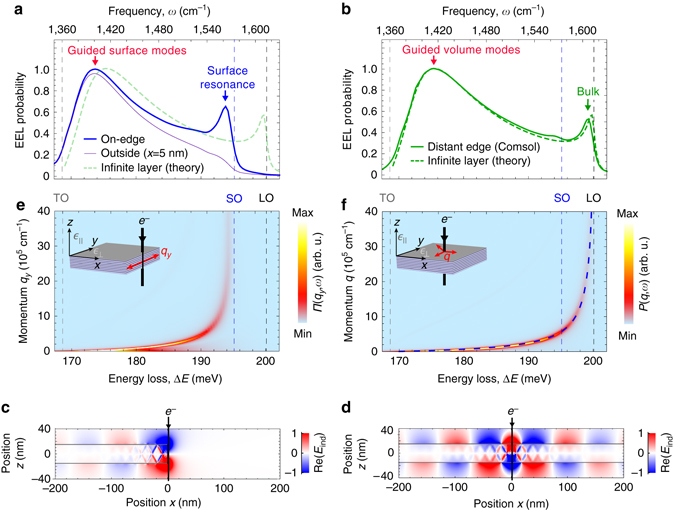
Volume vs. edge-localized hyperbolic polaritons. **a** EEL probability spectrum of 30-nm-thick semi-infinite slab for an on-edge electron trajectory (*blue*) and for a trajectory 5 nm outside the slab (*thin purple*) obtained using full-wave simulation (Comsol). **b** EEL probability spectrum for the beam passing through the h-BN slab away from the edge (off-edge trajectory) obtained using Comsol (*solid green*). *Dashed green* spectra in **a** and **b** are retarded theoretical calculations (see Supplementary Note 5) using Eq. (1). **c** Cross section of the induced electric fields (z-component) taken at *ω* = 1570 cm^−1^ for the on-edge electron trajectory. **d** Same as in **c** but for the off-edge electron trajectory. **e** Momentum dependent probability *П*(*q*_*y*_,*ω*) for the on-edge electron trajectory. **f** Retarded calculation of *P*(*q*_*y*_,*ω*) for a 30-nm-thick, laterally infinite h-BN layer (see Supplementary Note 5), i.e. the sum of contributions depicted in Fig. 2a–c, but with retardation included. *Dashed blue* curve marks the dispersion of M0-HPhP mode. *Insets* in **e** and **f** illustrate the electron trajectories and the polariton propagation directions. In all plots, the *vertical dashed lines* mark the TO, SO, and LO phonon energies

To understand the redshift of EEL spectra when the beam crosses the h-BN edge (see the area at the bottom flake edge in Fig. 1d), we numerically simulate (Comsol Multiphysics, see Methods for details) the energy loss spectra for the electron passing directly at the edge (on-edge trajectory, see inset in Fig. 5e) of a 30 nm thick, semi-infinite slab (thick blue curve in Fig. 5a). Interestingly, we find two major differences with respect to the spectrum of an infinite h-BN slab (solid green curve in Fig. 5b): (i) the loss peak due to the guided mode excitation (marked by a red arrow at 174 meV in Fig. 5a) is redshifted by 2 meV and (ii) the bulk loss peak near the LO energy (marked by a green arrow in Fig. 5b) is shifted to 194 meV (1570 cm^−1^, marked by a blue arrow in Fig. 5a). The lower energies of both peaks explain the redshift of the EEL spectra at the flake edge (Fig. 1d, bottom edge).

To understand the position of the peaks in the EEL probability spectra at the flake edge, we calculated the cross sections of the electric field **E**_ind_ (evaluated at the energy loss Δ*E* = 194.7 meV) induced by the electron beam for an on-edge and an off-edge (away from the edge through the slab, see inset in Fig. 5f) electron trajectory (Fig. 5c, d, respectively). For the off-edge electron trajectory, we observe a zig-zag field pattern inside the h-BN slab, revealing the typical HPhP rays (see Fig. 3a). Above and below the h-BN slab (|*z*| > 15 nm), we find the field oscillations that correspond to the M0-HPhP mode^29–31^. For the on-edge electron trajectory, we additionally observe a strongly localized field at the h-BN edge, which indicates the excitation of a surface polariton that propagates along the surface of the flake edge (along the *y*-axis)^12^.

We note that surface polaritons in vdW materials do not exist on surfaces parallel to the atomic layers, i.e., on the top and bottom surfaces of the flakes. However, they can exist at the flake edges, whose surfaces are parallel to the optical axis of the vdW crystal and exhibit a strong in-plane optical anisotropy^34, 35^. The surface polaritons on such surfaces exhibit a hyberbolic dispersion, and thus are called hyperbolic surface polaritons. Analogue to conventional surface polaritons, they can be identified by their dispersion that for large momenta asymptotically approaches the surface optical phonon (SO) energy given by the condition *ϵ*_⊥_(*ω*_SO_) = −1^12^.

In order to verify that indeed a surface polariton is excited at the flake edge, we study its dispersion. To this end, we analyze the momentum-dependent energy loss function *П*(*q*_*y*_,ω) (Fig. 5e), which was used to calculate the EEL probability ${\it{\Gamma }}\left( \omega \right) = \mathop {\smallint }\nolimits {\int{{\Pi}}}\left( {{q_y},\omega } \right){\rm d}{q_y}$ shown in Fig. 5a,b (see Methods for details). *П* is analogous to *P* in Eq. (1) and represents the probability of exciting a wave with momentum *q*_*y*_ parallel to the h-BN edge (see inset in Fig. 5e). We see that *П*(*q*_*y*_,*ω*) exhibits a pronounced maximum, which reveals the dispersion of a single dominant mode. For large momenta, it asymptotically approaches the energy of 195 meV (vertical blue dashed line in Fig. 5e), in sharp contrast to the dispersion of M0-HPhP mode (Fig. 5f) that tends toward the LO energy. For h-BN, this asymptote at 195 meV corresponds precisely to the SO energy^12^. We can thus conclude that the electron beam excites a hyperbolic surface phonon-polariton (HSPhP) propagating at the edge surface of the flake^11, 12, 36^. The peak in *Γ*(*ω*) at SO energy (marked by a blue arrow in Fig. 5a) can thus be regarded as the loss due to a surface (phonon) resonance^12^, i.e. resonant HSPhP excitation arising from a divergence of the local density of HSPhP states near the SO energy^20^. The EEL peak at 174 meV (red arrow in Fig. 5a) can be also attributed to the HSPhP excitation, however it originates from the excitation of the HSPhP mode that is guided along the flake edge (i.e. guided surface mode). The spectral position of this peak, *ω*_g,HSPhP_, is determined by the intersection of the line defined by Eq. (4) with the HSPhP mode dispersion, analogously to the energy loss due to guided volume mode excitation in the laterally infinite h-BN slab (see Fig. 3d, f). As the HSPhP mode dispersion has a steeper rise^12^ with energy compared to the M0-HPhP mode dispersion, the peak position *ω*_g,HSPhP_ = 174 meV is redshifted compared to the HPhP peak at *ω*_g,HPhP_ = 176 meV.

**Fig. 6:**
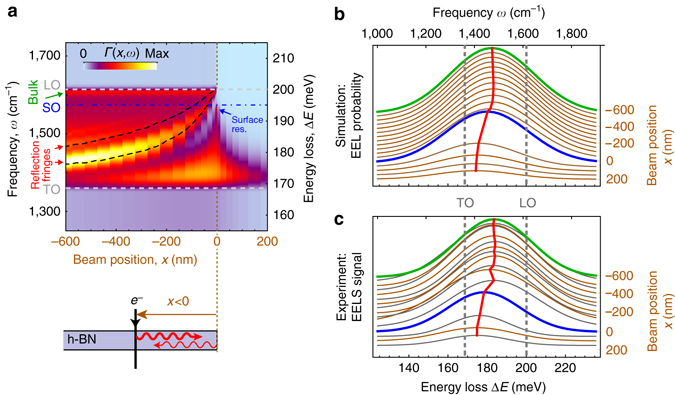
Electron energy loss spectra near flake edges. **a** Numerically calculated (Comsol) EEL probability spectra for 30-nm-thick semi-infinite h-BN slab as a function of distance to its edge. *Black dashed curves* trace the interference fringe maxima appearing due to reflection of the guided volume polaritons from the slab edge (schematically shown at the bottom) calculated using Eq. (6) with reflection phase *ϕ*_refl_ = *π*/2. **b** Spectra extracted from **a**, but after convolution according to Eq. (5). The spectra are offset vertically for better visibility. *Red line* marks the position of the peak maximum. *Blue spectrum* corresponds to the convolution of the solid blue spectrum in Fig. 5a. *Vertical dashed lines* mark the TO and LO phonon energies. **c** Gaussian fits to the experimental STEM-EELS spectra for different beam positions near the edge of the 30 nm thick part h-BN flake. The spectra are collected along the *orange line* marked in Fig. 1a (see Methods). *Red line* marks the position of the peak maximum, *ω*_0_

To obtain the complete picture of energy loss at both sides of the flake edge, we numerically simulate EEL probability spectra *Γ*(*x*,*ω*) for beam positions *x* on and outside the 30-nm-thick, semi-infinite h-BN slab (Fig. 6a, top panel). For the electron passing through the h-BN flake (*x* < 0), these spectra exhibit a small bulk loss peak at the LO energy (marked by the green arrow), which is consistent with our previous theoretical analysis (see green and dashed green curve in Figs. 3f and 5b, respectively). However, instead of a single peak due to the excitation of the M0-HPhP mode (red and dashed green curves in Figs. 3f and 5b, respectively), a set of fringes appears (marked by red arrows in Fig. 6a), whose positions depends on the distance to the flake edge. These fringes can be understood as an interference pattern caused by the electron beam-launched M0-HPhP mode, which is reflected at the h-BN flake edge^29, 30, 37^ (illustrated by the bottom schematics in Fig. 6a). This reflection can be phenomenologically introduced into our theory by adding a cos(2*q*|*x*| + *ϕ*_refl_) in Eq. (1):

6 $${\it{\Gamma }}\left( {x,\omega } \right) = {\int}_{\!\!\!\!\! 0}^{{q_{\rm{c}}}} {P\left( {q,\omega } \right)\left[ {1 + \cos \left( {2q\left| x \right| + {\phi _{{\rm{refl}}}}} \right)} \right]{\rm d}q} $$

where 2*q*|*x*| is the propagation phase acquired by an HPhP upon the propagation toward the edge and back;^37^ *ϕ*_refl_ is the phase acquired upon reflection^38^. We calculated *Γ*(*x*,*ω*) according to Eq. (6) and plotted its maxima as black dashed traces in Fig. 6a. We find a great agreement of these analytical lines with the fringe maxima obtained in our Comsol simulations (color plot in Fig. 6a), proving that these fringes are due to HPhP interference.

For aloof beam trajectories (*x* > 0 in Fig. 6a), neither the bulk loss peak near LO nor the surface phonon resonance peak near the SO energy (marked by green and blue arrows, respectively) appear in the simulated EEL spectra. These peaks originate from the high-momentum excitations in the sample (see Supplementary Note 4), which are highly localized and, therefore, are less accessible by aloof beams. As a result, only the peak associated with the excitation of guided modes is present in the aloof beam spectra. Remarkably, this peak matches the one in the on-edge spectrum (see the thin purple spectrum in Fig. 5a for better comparison), signifying that near the edges of vdW materials the electron predominantly excites the guided hyperbolic surface polariton modes and not the volume-propagating HPs.

To verify our analysis of the EEL near the edges of vdW materials, we convolve the simulated spectra *Γ*(*x*,*ω*) in Fig. 6a with a 46 meV Gaussian function (to mimic the experimental spectral resolution) and compare the results (Fig. 6b) with the experimental STEM-EELS spectra (Fig. 6c) of h-BN collected along the orange line in Fig. 1a (see Methods for details). We see that the peak position *ω*_0_ of the convolved spectra (red curve in Fig. 6b) is in excellent agreement with that in our experiment (red curve in Fig. 6c), which supports the validity of our theoretical understanding. We also see that the current spectral resolution of our STEM-EELS is not sufficient yet to directly observe the interference fringes for electron trajectories inside the slab (*x* < 0 in Fig. 6a), which can be resolved by near-field optical microscopy^29, 30, 37^. However, we expect that the mapping of the HP interferences fringes becomes possible in the near-future with specialized low-energy STEM-EELS instruments that reach an energy resolution below 10 meV^14, 39^.

Interestingly, we see that the position of the h-BN peak is not constant for aloof beam spectra (*x* > 0 region in Fig. 6b, c), but gradually redshifts with distance to the h-BN edge. This redshift can be explained by the stronger spatial decay of EEL probability at higher energies, which is clearly visible in Fig. 6a (for *x* > 0)^40^). Typically, this decay is attributed purely to the Coulomb potential of the moving electron described by *K*_0_(2*ω*|*x*|/*v*), where *K*_0_ is the modified Bessel function of the 2-nd kind that decays exponentially for large arguments^14, 20, 41–44^. In other words, the signal decay constant, *κ*, is assumed to linearly depend on the energy loss: *κ* = 2*ω*/*v*. However, near the edges of polaritonic materials we expect *κ* to also depend on the extent of the field of the dominant polariton mode (e.g., that of HSPhP in the case of polar vdW materials), which strongly depends on the polariton mode momentum. This dependence could introduce another, momentum-dependent scale for the aloof signal delocalization in EEL spectroscopy near vdW material edges. A detailed study of this interesting topic would go beyond the scope of this work and will be subject of future work.

## Discussion

We enabled a standard STEM-EELS system to detect low-energy excitations in vdW materials and used it to spatially map phononic excitations of h-BN at mid-IR frequencies. We demonstrated that the electron beam primarily probes the hyperbolic polaritons and not the bulk phonons, which is responsible for the unusual positions of the spectral peaks. We further showed that near the flake edges the electron beam primarily excites a hyperbolic surface polariton propagating along the edge surface of the vdW material—the finding being important for the interpretation of spectra in aloof spectroscopy employed for avoiding beam damage to fragile layered structures^44, 45^. Our findings are supported by a classical dielectric theory of EEL in strongly anisotropic materials that can be applied to studies of phononic, plasmonic, and excitonic media.

The dominance of hyperbolic polaritons in EEL spectra of vdW materials makes STEM-EELS potentially suitable for the investigation of optical/polaritonic properties of such materials, with this work serving as a foundation for such investigations. With ongoing developments in monochromator and electron gun designs^39^—ZLP being already as narrow as 10 meV in state-of-the-art specialized instruments^14^—we expect a significant improvement of the STEM-EELS spectral resolution in the near future, allowing for correlative studies of polaritons and structural properties in vdW materials, and turning the technique into a powerful complement to optical near-field methods.

## Methods

### Sample fabrication

We first performed mechanical exfoliation of commercially available h-BN crystals (HQ graphene Co., N2A1) using blue Nitto tape (Nitto Denko Co., SPV 224P). Then, we performed a second exfoliation of the h-BN flakes from the tape onto a transparent polydimethyl-siloxane stamp. After that, via optical inspection of the stamp, a flake with desired topography was identified and transferred onto a suspended 15-nm-thick transmission electron microscopy (TEM) membrane (ultrathin TEM grid, Ted Pella, Inc.) using the dry transfer technique^46, 47^. According to the manufacturer specifications these membranes are nonstoichiometric amorphous oxi-nitride films (i.e. are slightly O-doped to reduce the mechanical strains) with a general stoichiometry given by SiN_x_O_y_. The h-BN flake was freshly exfoliated and no sample contamination was observed during our studies.

We note that the flake surfaces might contain adatoms. However, we do not expect them to significantly affect the HP modes (i.e., cause significant shifts of EEL peaks). This is because the delocalization scale of a polariton mode is much larger than the atomic scale, thus adatoms can only introduce a small perturbation to the results presented in this work and can be safely ignored.

### Enhancing beam monochromaticity and the spectral resolution

EELS measurements were performed as STEM spectrum imaging at 60 kV with a low-base Titan 60–300 TEM (FEI Co, Netherlands) equipped with a high brightness electron gun (x-FEG), electron beam monochromator and Gatan Quantum 965 imaging filter (GIF). The overall performance of (S)TEM-EELS system is mainly determined by two factors: the energy spread of electrons in the primary electron beam and the energy resolution of electron energy detection system, i.e., GIF.

To improve the monochromaticity (i.e., energy spread) of the primary electron beam we decreased the potential of the monochromator (operated with a standard excitation of 1–1.2) from a typical 3000 V down to 700 V while keeping the gun lens potential at around standard 700 V. This way we moved the first gun crossover down from the accelerator to the plane close to the energy selecting slit. As a result, the crossover occurs for the electrons with a maximum speed for a given high tension. This dramatically decreases the energy spread broadening due to electron Coulomb interaction and, therefore, improves the electron beam monochromaticity.

To improve the energy resolution of the EELS detection system, we reduced the ZLP broadening due to a finite size of the GIF channel (usually estimated as three times the energy of a single channel) by implementing a special dispersion of 1.7 meV/channel (as measured by electrostatic drift tube of the GIF). Such dispersion is more than five times better than the smallest dispersion available in the majority of commercial GIFs, reducing the ZLP broadening in the GIF by at least 25 meV. To further minimize the effect of aberrations (that negatively affect the EELS detection system resolution), we operated our microscope in microprobe STEM mode with a probe semi-convergence angle of about 1 mrad and extremely low camera length to obtain the collection angle of about 8 mrad.

The combination of system modifications described above, allowed us to reach on a regular basis an energy resolution of about 45–50 meV per 1 ms of acquisition time, and 38 meV per 1 ms at peak performance. These results are rather reproducible and agree well with theoretical predictions^48^. Testing of our approach on another microscope (high-base Titan, 966 GIF) showed the same energy resolution also at 80 keV.

### Spectra acquisition, noise reduction, and filtering

Using the modified STEM-EELS system (see above) we have acquired a hyperspectral image of our sample, which contains an EEL spectrum (with 1 ms acquisition time per spectrum) at each spatial pixel (the beam is scanned with a step of 9.4 nm). Part of the image (about 1/3 of the total acquisition area directly above the area shown in Fig. 1a, d) was obtained with the electron beam switched off. The EELS signal from this area contains only the dark noise of the spectrometer. We averaged this dark noise among all spatial pixels (separately for each energy channel) and then subtracted it from every measured spectrum. Importantly, this subtraction was performed channel-by-channel and prior to the energy axis calibration (i.e., assigning zero energy to the channel with the maximum signal count), because the dark noise is channel specific.

The distribution of all spatial pixels was then evaluated based on the FWHM of the corresponding ZLP, which shows additional (to the main width distribution) peaks. These peaks could be attributed to mechanical vibrations and electromagnetic interference. We thus discard all pixels with ZLP width outside the main distribution and replace their spectra by interpolation from the nearest neighbors.

### Processing and analysis of experimental EEL spectra

After the dark noise subtraction and pixels filtering (as described above), each EEL spectrum was normalized to its maximum. The signal from the bare membrane, which we regard as ZLP, was then averaged in the region marked by magenta line in Fig. 1a to suppress the noise. This averaged ZLP was then subtracted from each spectrum, yielding the h-BN inelastic scattering spectrum (i.e., energy loss to h-BN crystal lattice vibrations). To avoid possible drifts along the slow scan direction, the ZLP averaging and subtraction was performed separately for each horizontal line (that are aligned with the fast scan axis here). The h-BN inelastic scattering spectrum in the range from 140 to 220 meV was then fitted with a Gaussian, returning the best fit position of the peak maximum *ω*_0_. Finally, all the points with signal level below 2.5 × 10^−4^ threshold or with irregular fits (*ω*_0_ outside of the upper Reststrahlen band or with FWHM < 40 meV, i.e., below system spectral resolution) were filtered out and depicted in black in the map presented in Fig. 1d.

We note that fitting of the h-BN spectra by a single Gaussian is justified by the excellent agreement between the experimental data and the fit (see Fig. 4d, for example). This excellent agreement can be explained as follows. Since the major loss peak (related to the excitation of HPhPs) is much narrower than the ZLP, the convolution Eq. (5) yields a peak with the same shape as that of ZLP. The latter is a Gaussian to a very good approximation. The narrow peak related to the bulk loss contributes only 6% to the total spectral loss (calculated by integrating *Γ*_bulk_(*ω*) within two FWHM around its maximum at LO). Convolution thus yields a slight spectral blueshift without significant distortion of the Gaussian shape.

### h-BN film thickness estimation

After performing STEM-EELS, the h-BN sample was scanned using AFM yielding a topography image. The film thickness along the flake edges was determined through the step height measured as the AFM tip crosses from the membrane to the h-BN. To estimate the film thickness away from the edges, we associate the high-angle annular dark-field (HAADF) image contrast with the thickness, which can be done due to their monotonous interdependence. We then identify plateaus of the same contrast (marked by dashed white lines in Fig. 1a) and read off their thicknesses from the height at the plateaus’ edges. This procedure yields the thicknesses of about 2, 15, and 30 nm for the blue, red, and green areas marked in Fig. 1a, respectively.

### Line profile of the h-BN EEL spectrum across the flake edge

The peak position in Fig. 6c was obtained by taking the h-BN spectra along the orange line marked in Fig. 1a. For each vertical position, spectra were averaged horizontally (seven pixels on both sides) and fitted with a Gaussian (curves in Fig. 6c), yielding the depicted profile of peak position, *ω*_0_.

### Full-wave numerical simulations of the EEL probability

The flake edge breaks the cylindrical symmetry, which prohibits the direct calculation of EEL according to Eq. (1). Therefore, EEL probability spectra near the h-BN edge (shown in Figs. 5 and 6) were simulated using a finite element method (Comsol Multiphysics) based on solving Maxwell’s equations in the frequency domain. The moving electron (represented by a linear current) acts as a source, creating the electromagnetic field in the system. The calculation for each frequency was performed twice: with and without the dielectric environment (sample), preserving the same mesh. Their difference yields **E**_ind_, which can be integrated along the electron trajectory to find *Γ*(*ω*)^49, 50^:

7 $${\it{\Gamma }}\left( {x,\omega } \right) = \frac{e}{{\pi \hbar \omega }}{\rm R}{\rm e} \,\left[ {{\int}_{_{\!\!\!\!\!\!\! - \infty }}^{^\infty } {{\rm{d}}z\,{{\hat {\bf z}}} \cdot {{{\bf E}}_{{{\rm ind}}}}} \left( {x,y,z;\omega } \right)\,{\rm{exp}}\left( { - {\rm{i}}\frac{{\omega z}}{v}} \right)} \right],$$

where *x* and y describe the lateral position of the electron across and along the edge, respectively (note that the energy loss is *y*-independent due to translational invariance of the system along the edge). Instead of directly solving for the induced field, we rewrite Γ(*x*,*ω*) using the Fourier transform (with respect to *y*) of ${{\bf{E}}_{\rm ind}}\left( {x,y,z;\omega } \right) = \frac{1}{\pi }{\int}_{_0}^{^\infty } {{\rm{d}}{q_y}{{{{\tilde {\bf E}}}}_{{\rm{ind}}}}} \left( {x,z;{q_y},\omega } \right)\exp \left( {{\rm{i}}{q_y}y} \right).$ By setting $y = 0$ (without loss of generality) we can write:

8 $${\it{\Gamma }}\left( {x,\omega } \right) = {\int}_{\!\!\!\!_0}^{^\infty } {{\rm d}{q_y}{\it{\Pi}}\left( {{q_y},\omega } \right)} ,$$

where ${\it{\Pi}}\left( {{q_y},\omega } \right) = \frac{e}{{{\pi ^2}\hbar \omega }}{\rm R}{\rm e} \,\left[ {{\int}_{_{\!\!\! - \infty }}^{^\infty } {{\rm{d}}z\,{{\hat {\bf z}}}} \cdot {{{{\tilde {\bf E}}}}_{{{\rm ind}}}}\left( {x,z;{q_y},\omega } \right)\exp \left( { - {{i}}\frac{{\omega z}}{v}} \right)} \right]$. This allows for replacing a single problem of finding **E**_ind_ in three dimensions with a set of 2D problems of finding ${{{\tilde {\bf E}}}_{{\rm{ind}}}}$ as a function of *x* and *z* for fixed *q*_*y*_. We, thus, performed the calculations for discrete *q*_*y*_ values up to the cutoff momentum *q*_c_ and added up the individual contributions to find the total probability. Doing so, allowed for maintaining the same *q*_c_ for different beam positions *x*, resulting in consistent calculation of the bulk loss. In addition, it allowed for the direct visualization of *П*(*q*_*y*_,*ω*) in Fig. 5e.

We note that in the theoretical study presented in the main text, we did not consider the 15 nm thick TEM membrane that supports the h-BN flake, as it does not exhibit phonons and phonon polaritons within or close to the h-BN Reststrahlen band. The membrane only acts as a dielectric substrate with a refractive index of about 2^51^. It leads to slight redshifts (about 0.5 meV) of the h-BN HPhP peaks (see Supplementary Note 6 and Supplementary Fig. 6), which are small compared to the spectral shifts observed in this work and are also within the experimental uncertainty of our EELS detector.

### Data availability

The data that support the findings of this study are available from the corresponding author upon reasonable request.

## Electronic supplementary material

Supplementary Information
