# Supplementary material for: Probing low-energy hyperbolic polaritons in van der Waals crystals with an electron microscope
Source: Nat Commun. 2017 Jul 21;8:95. doi: 10.1038/s41467-017-00056-y (PMC5522439; doi:10.1038/s41467-017-00056-y)
Supplement: Supplementary file 1 — Supplementary Information [file 41467_2017_56_MOESM1_ESM.pdf]

File Name: Supplementary Information
Description: Supplementary Figures, Supplementary Notes and Supplementary References

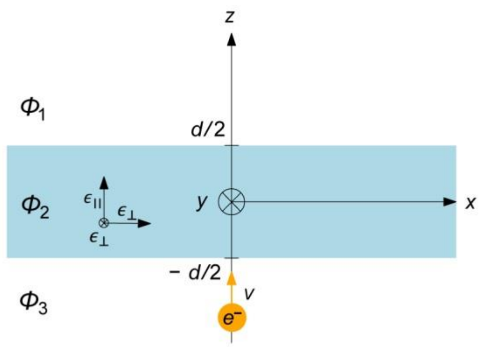


**Supplementary Figure 1: System geometry considered in the analytical calculation of the electron energy loss.**

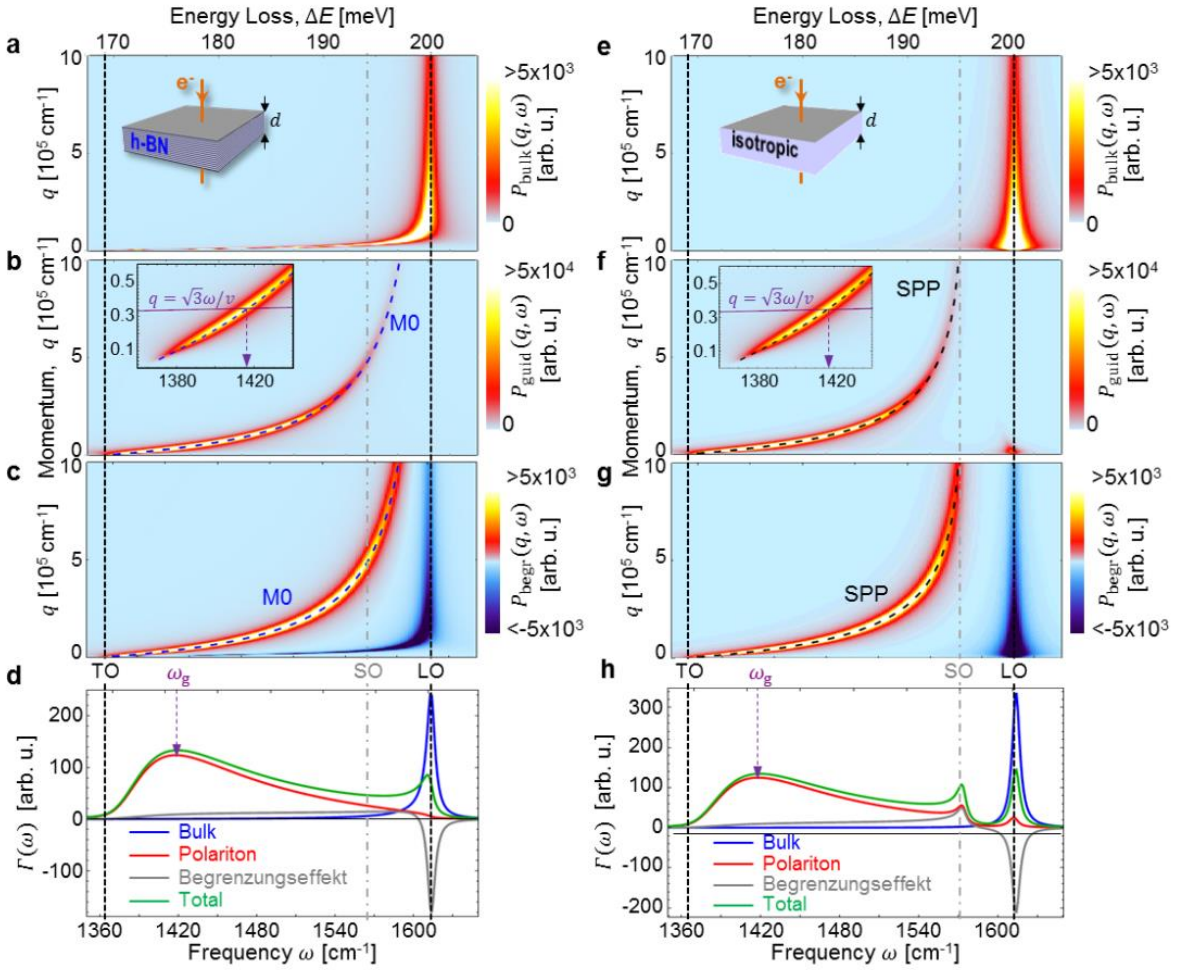


**Supplementary Figure 2: Theoretical EEL spectrum for an anisotropic (a-d) and isotropic (e-h) homogeneous slabs.** Left column shows the same as in Fig. 3c-f of the main text, namely $P_{\text{bulk}}(q,\omega)$ (**a**), $P_{\text{guid}}(q,\omega)$ (**b**), $P_{\text{begr}}(q,\omega)$ (**c**) and their contributions to $\Gamma(\omega)$ (**d**) calculated for 30 nm thick h-BN slab and 60 keV electron energy. Right column shows the same calculations but for the isotropic slab with permittivity $\epsilon_{||} = \epsilon_{\perp}$. The black dashed curves in **f** and **g** depict the dispersion of the (guided) SPP mode calculated according to Eq. (27). Inset in **f** shows an unsaturated blow up view of the surface contribution at low momenta $q$ showing the line $q = \sqrt{3}\omega/v$ (purple) representing the maximum of momentum transfer from electron to the guided mode; dashed arrow marks the energy at which this line intersects with the SPP dispersion curve. **h,** EEL probabilities, $\Gamma$, obtained by integration (over momentum) of individual contributions shown in **e**, **f**, and **g** and their sum. The vertical purple arrow marks the same energy as in the inset of **f**. On all plots the vertical dashed lines mark the TO and LO phonon energies; vertical dot-dashed line shows the location of the surface phonon energy (corresponds to $\epsilon_{\perp} = -1$ here).

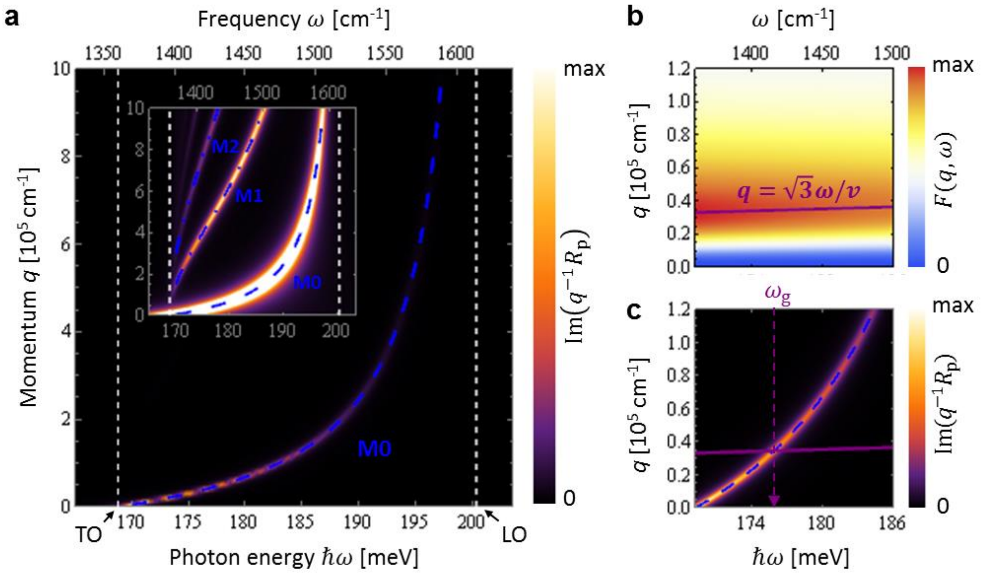


**Supplementary Figure 3: EEL due to HP excitation. a,** $\mathrm{Im}\left[q^{-1}R_\mathrm{p}(q,\omega)\right]$ (false color) and the dispersion of the M0-HPhP mode found using Eq. (27) for 60 keV electron passing through 30 nm thick h-BN slab. The vertical dashed lines mark TO and LO energies. The inset shows the same plot but with color scale saturated to show the higher order guided modes. **b,** Function $F(q,\omega)$ that could be interpreted as the probability of HP excitation at momentum $q$ and energy $\omega$. **c,** Zoom in into the low momentum part of the plot in **a**. Purple line in **b** and **c** marks the maximum of $F$ in $(q,\omega)$ space. The vertical dashed line in **b** marks the energy at which the electron line crosses the M0-HPhP dispersion, and which determines the peak position in $\Gamma_\mathrm{guid}(\omega)$.

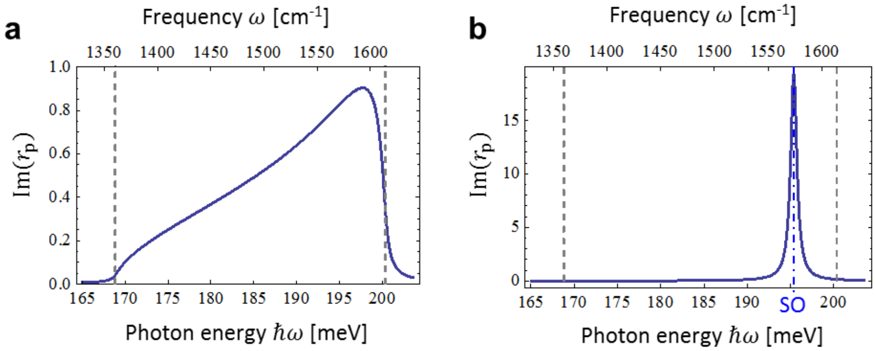


**Supplementary Figure 4: The reflection coefficient (imaginary part) from a thin slab at large momenta.** Imaginary part of the quasistatic refection coefficient, $r_p$, (to which $R_p$ reduced at $q \to \infty$) for a single interface with the anisotropic (**a**) and isotropic (**b**) media. The isotropic case was calculated by taking $\epsilon_{||} = \epsilon_{\perp}$. Left and right vertical dashed lines mark TO and LO phonon energies, respectively; the blue dash-dotted line in **b** marks the SO energy.

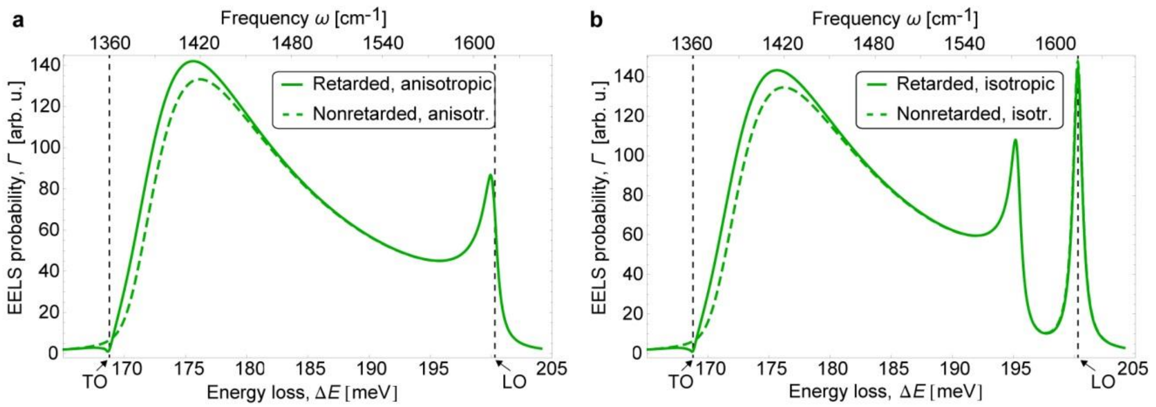


**Supplementary Figure 5: Retardation effects.** Comparison of EEL spectra for the anisotropic (**a**) and isotropic (**b**) retarded/non-retarded calculations obtained for an electron with energy 60 keV ($v = 0.446\ c$) that passes through a 30 nm thick slab. For the isotropic case, the response was characterized by $\epsilon_{\perp}$ in all directions. The cutoff is $q_{\mathrm{c}} = 0.082$ a.u., which corresponds to the experimental value of 8 mrad for aperture collection angle.

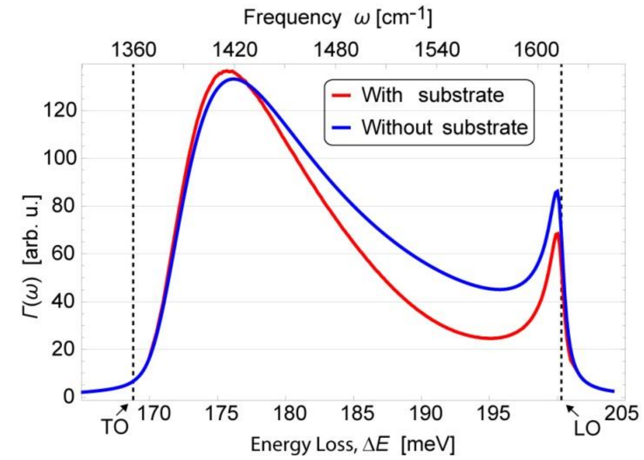


**Supplementary Figure 6: Influence of the substrate.** Comparison of non-retarded EEL spectra with and without a thin isotropic substrate of 15 nm thickness and constant permittivity $\epsilon_{\mathrm{sub}} = 4$. The electron energy is 60 keV ($v = 0.446\ c$) and the h-BN layer thickness is 30 nm. The cutoff momentum is $q_{\mathrm{c}} = 0.082$ a.u.

## Supplementary Note 1: Analytical calculations of EELS probability

The energy lost by an elecxtron can be found as its work $W$ against the electromagnetic field $\mathbf{E}_{\mathrm{ind}}$ that it induces[1–3]. Assuming a straight line electron trajectory (non-recoil approximation) parallel to z-axis (Supplementary Figure 1), we write:

$$W = -e \int_{-\infty}^{\infty} \mathrm{d}z\, \hat{\mathbf{z}} \cdot \mathbf{E}_{\mathrm{ind}}(\mathbf{r}_{\mathrm{t}}(t), t). \quad (1)$$

By utilizing the Fourier transform and cylindrical symmetry of the sample, it can be written as:

$$W = -\frac{2e}{(2\pi)^3} \mathrm{Re}\left[\int_0^{\infty} \mathrm{d}\omega \int_0^{\infty} \mathrm{d}q\ 2\pi q \int_{-\infty}^{\infty} \mathrm{d}z\, \hat{\mathbf{z}} \cdot \mathbf{E}_{\mathrm{ind}}(q, z, \omega) \exp\left(\frac{-\mathrm{i}\omega z}{v}\right)\right], \quad (2)$$

where $e$ is the elementary charge, $v$ is the electron speed and $q$ is the momentum in the plane perpendicular to the electron trajectory $\mathbf{r}_{\mathrm{t}}(t) = (x_t, y_t, vt)$ (without the loss of generality $x_t$ and $y_t$ could be set to zero). The probability $\Gamma(\omega)$ for the electron to lose energy $\hbar\omega$ can be related to $W$ as:

$$W = -\int_0^{\infty} \mathrm{d}\omega\, \hbar\omega\, \Gamma(\omega). \quad (3)$$

Eq. (1) of the main text can be obtained by solving for $\Gamma$ in Eqs. (2) and (3) and defining the momentum dependent function $P(q, \omega)$:

$$P(q, \omega) = \frac{eq}{2\pi^2 \hbar\omega} \mathrm{Re}\left[\int_{-\infty}^{\infty} \mathrm{d}z\, \hat{\mathbf{z}} \cdot \mathbf{E}_{\mathrm{ind}}(q, z, \omega) \exp\left(\frac{-\mathrm{i}\omega z}{v}\right)\right]. \quad (4)$$

Hence, to calculate the loss probability, we have to find the electric field induced by the electron in the sample.

## Supplementary Note 2: Nonretarded approximation

In the non-retarded approximation, the electric field can be expressed through the scalar potential, $\Phi$, as $\mathbf{E} = -\boldsymbol{\nabla}\Phi$. Writing the Gauss's law using this potential yields:

$$\boldsymbol{\nabla} \cdot [\hat{\epsilon}\, \boldsymbol{\nabla}\Phi(\mathbf{r}, t)] = \frac{\rho_{\mathrm{f}}}{\epsilon_0}. \quad (5)$$

where $\epsilon_0$ is the vacuum permittivity, $\rho_{\mathrm{f}} = e\delta[\mathbf{r} - \mathbf{r}_{\mathrm{t}}(t)]$ is the free charge density corresponding to the moving electron and $\hat{\epsilon} = \mathrm{diag}(\epsilon_{\perp}, \epsilon_{\perp}, \epsilon_{||})$ is the dielectric tensor. Such tensorial treatment of dielectric permittivity is essential for proper description of EEL in vdW materials due to their strong uniaxial anisotropy.

By Fourier transforming Eq. (5) with respect to $x$ and $y$ and utilizing the cylindrical symmetry we obtain

$$\epsilon_{||} \frac{\partial^2 \Phi(q,z,\omega)}{\partial z^2} - q^2 \epsilon_{\perp} \Phi(q, z, \omega) = \frac{e}{v\, \epsilon_0} \exp\left(\frac{\mathrm{i}\omega z}{v}\right). \quad (6)$$

In each of the regions marked in Supplementary Figure 1, the potential can be represented as a sum $\Phi_k = \Phi_{\mathrm{F},k} + \Phi_{\mathrm{B},k}$, where $\Phi_{\mathrm{F},k}$ is the free-space part that corresponds to the solution of Eq. (6) in unbounded medium:

$$\Phi_{\mathrm{F},k} = -\frac{ev\exp(\mathrm{i}\omega z/v)}{\epsilon_0(\epsilon_{||}\omega^2+\epsilon_{\perp}q^2v^2)}, \tag{7}$$

and $\Phi_{\mathrm{B},k}$ is the part that arises from the presence of boundaries. In each region, it can be written as:

$$\Phi_{\mathrm{B},k} = A_k\exp\left(q\sqrt{\epsilon_{\perp}/\epsilon_{||}}z\right) + B_k\exp\left(-q\sqrt{\epsilon_{\perp}/\epsilon_{||}}z\right) \tag{8}$$

For isotropic regions ($k = 1,3$) we simply set $\epsilon_{\perp} = \epsilon_{||}$. The coefficients $A_k$ and $B_k$ in Eq. (8) are found by solving the standard boundary value problem[4]. Requirement of finiteness of the potential at infinity immediately gives $A_1 = B_3 = 0$ and by imposing the continuity of the potential and its $z$ derivative at the interfaces we obtain the remaining coefficients.

$\mathbf{E}_{\mathrm{ind}}$ is the difference between the field produced by the electron in the presence of the sample and that in vacuum, and can be found as $\mathbf{E}_{\mathrm{ind}} = -\boldsymbol{\nabla}\Phi^{\mathrm{ind}}$ with the induced potential defined as:

$$\Phi_k^{\mathrm{ind}} = \Phi_k - \Phi_0, \tag{9}$$

Here $\Phi_0$ is the solution of Eq. (6) for the electron in vacuum in the absence of sample, i.e. $\Phi_0 = \Phi_{\mathrm{F},k}$ for $k = 1,3$ assuming vacuum in the corresponding regions. From Eq. (9) and (7), we immediately see that the induced potential outside has only the free-space component, whereas inside both the free-space and boundary parts remain.

Using the induced electrostatic potential $\Phi^{\mathrm{ind}}$, $P(q,\omega)$ in Eq. (4) can be written as:

$$P(q,\omega) = \frac{eq}{2\pi^2\hbar v}\mathrm{Im}\left[\int_{-\infty}^{\infty}\mathrm{d}z\,\Phi^{\mathrm{ind}}(q,z,\omega)\exp\left(\frac{-\mathrm{i}\omega z}{v}\right)\right]. \tag{10}$$

Naturally, the integral splits into three contributions. The first part is coming from the integration along the parts of the trajectory outside the slab:

$$P_{\mathrm{guid}}(q,\omega) = \frac{eq}{2\pi^2\hbar v}\mathrm{Im}\left[\int_{-\infty}^{-d/2}\mathrm{d}z\,\Phi_1^{\mathrm{ind}}(q,z,\omega)\exp\left(\frac{-\mathrm{i}\omega z}{v}\right) + \int_{d/2}^{\infty}\mathrm{d}z\,\Phi_3^{\mathrm{ind}}(q,z,\omega)\exp\left(\frac{-\mathrm{i}\omega z}{v}\right)\right] = \frac{eq}{2\pi^2\hbar}\mathrm{Im}\left[A_3\frac{\exp[-d/2(q-\mathrm{i}\omega/v)]}{qv-\mathrm{i}\omega} + B_1\frac{\exp[-d/2(q+\mathrm{i}\omega/v)]}{qv+\mathrm{i}\omega}\right], \tag{11}$$

and is related to the excitation of the guided modes in the slab, as shown later. The other two contributions come from the integration over the part of the trajectory penetrating the slab and relate to the free-space and boundary contributions to the induced potential:

$$P_{\mathrm{in}}(q,\omega) = \frac{eq}{2\pi^2\hbar v}\mathrm{Im}\left[\int_{-d/2}^{d/2}\mathrm{d}z\,\Phi_2^{\mathrm{ind}}(q,z,\omega)\exp\left(\frac{-\mathrm{i}\omega z}{v}\right)\right] = P_{\mathrm{bulk}}(q,\omega) + P_{\mathrm{begr}}(q,\omega). \tag{12}$$

The free-space part gives rise to the bulk loss

$$P_{\mathrm{bulk}}(q,\omega) = \frac{eq}{\pi^2\hbar}\mathrm{Im}\left[\frac{ed}{2\epsilon_0}\left(\frac{1}{\omega^2+q^2v^2} - \frac{1}{\epsilon_{||}\omega^2+\epsilon_{\perp}q^2v^2}\right)\right], \tag{13}$$

whereas the boundary part is responsible for the begrenzungseffekt

$$P_{\mathrm{begr}}(q,\omega)=\frac{eq}{\pi^2\hbar}\ \mathrm{Im}\left[\sqrt{\epsilon_{||}}\left(\frac{A_2\sin\left(\frac{\omega d}{2v}+\mathrm{i}dq\sqrt{\epsilon_{||}/\epsilon_{\perp}}/2\right)}{\omega\sqrt{\epsilon_{||}}+\mathrm{i}qv\sqrt{\epsilon_{\perp}}}+\frac{B_2\sin\left(\frac{\omega d}{2v}-\mathrm{i}qd\sqrt{\epsilon_{||}/\epsilon_{\perp}}/2\right)}{\omega\sqrt{\epsilon_{||}}-\mathrm{i}qv\sqrt{\epsilon_{\perp}}}\right)\right]. \quad (14)$$

The coefficients $A_{2,3}$ and $B_{1,2}$ read:

$$A_2=-\frac{t_{\mathrm{c}}\exp\left(\frac{qd}{2}\sqrt{\epsilon_{\perp}/\epsilon_{||}}-\frac{\mathrm{i}\omega d}{2v}\right)}{\left(1-r_{\mathrm{p}}^2\exp\left(-2d\sqrt{\epsilon_{\perp}/\epsilon_{||}}q\right)\right)q}\left[\left(-r_{\mathrm{p}}\exp\left(-2qd\sqrt{\epsilon_{\perp}/\epsilon_{||}}\right)+\exp\left(-qd\sqrt{\epsilon_{\perp}/\epsilon_{||}}+\frac{\mathrm{i}\omega d}{v}\right)\right)G+\left(r_{\mathrm{p}}\exp\left(-2qd\sqrt{\epsilon_{\perp}/\epsilon_{||}}\right)+\exp\left(-qd\sqrt{\epsilon_{\perp}/\epsilon_{||}}+\frac{\mathrm{i}\omega d}{v}\right)\right)Hq\right], \quad (15)$$

$$B_2=\frac{t_{\mathrm{c}}\exp\left(\frac{qd}{2}\sqrt{\epsilon_{\perp}/\epsilon_{||}}-\frac{\mathrm{i}\omega d}{2v}\right)\left[\exp\left(-qd\sqrt{\epsilon_{\perp}/\epsilon_{||}}\right)(G-Hq)-r_{\mathrm{p}}\exp\left(-2qd\sqrt{\epsilon_{\perp}/\epsilon_{||}}\right)\exp\left(\frac{\mathrm{i}\omega d}{v}\right)(G+Hq)\right]}{\left(1-r_{\mathrm{p}}^2\exp\left(-2qd\sqrt{\epsilon_{\perp}/\epsilon_{||}}\right)\right)q}, \quad (16)$$

$$A_3=\frac{t_{\mathrm{c}}\exp\left(\frac{qd}{2}-\frac{\mathrm{i}\omega d}{2v}\right)}{\left(1-r_{\mathrm{p}}^2\ \exp\left(-2qd\sqrt{\epsilon_{\perp}/\epsilon_{||}}\right)\right)q}\left[\sqrt{\epsilon_{||}\epsilon_{\perp}}\left(1-r_{\mathrm{p}}\exp\left(-2qd\sqrt{\epsilon_{\perp}/\epsilon_{||}}\right)-2\,t_{\mathrm{c}}\exp\left(-qd\sqrt{\epsilon_{\perp}/\epsilon_{||}}+\frac{\mathrm{i}\omega d}{v}\right)\right)Hq+\left(1+r_{\mathrm{p}}\exp\left(-2qd\sqrt{\epsilon_{\perp}/\epsilon_{||}}\right)-2\,t_{\mathrm{c}}\sqrt{\epsilon_{||}\epsilon_{\perp}}\exp\left(-dq\sqrt{\epsilon_{\perp}/\epsilon_{||}}+\frac{\mathrm{i}\omega d}{v}\right)\right)G\right], \quad (17)$$

$$B_1=\frac{t_{\mathrm{c}}\exp\left(\frac{qd}{2}+\frac{\mathrm{i}\omega d}{2v}\right)}{\left(1-r_{\mathrm{p}}^2\ \exp\left(-2qd\sqrt{\frac{\epsilon_{\perp}}{\epsilon_{||}}}\right)\right)q}\left[\sqrt{\epsilon_{||}\epsilon_{\perp}}\left(1-r_{\mathrm{p}}\exp\left(-2qd\sqrt{\epsilon_{\perp}/\epsilon_{||}}\right)-2\,t_{\mathrm{c}}\exp\left(-qd\sqrt{\epsilon_{\perp}/\epsilon_{||}}-\frac{\mathrm{i}\omega d}{v}\right)\right)Hq-\left(1+r_{\mathrm{p}}\exp\left(-2qd\sqrt{\epsilon_{\perp}/\epsilon_{||}}\right)-2t_{\mathrm{c}}\sqrt{\epsilon_{||}\epsilon_{\perp}}\exp\left(-qd\sqrt{\epsilon_{\perp}/\epsilon_{||}}-\frac{\mathrm{i}\omega d}{v}\right)\right)G\right], \quad (18)$$

$$H=\frac{ev}{\epsilon_0}\left(\frac{1}{\omega^2+q^2v^2}-\frac{1}{\epsilon_{||}\omega^2+\epsilon_{\perp}q^2v^2}\right),\qquad G=\frac{\mathrm{i}e\omega}{\epsilon_0}\left(\frac{1}{\omega^2+q^2v^2}-\frac{\epsilon_{||}}{\epsilon_{||}\omega^2+\epsilon_{\perp}q^2v^2}\right) \quad (19)$$

$$r_{\mathrm{p}}=\frac{\sqrt{\epsilon_{||}\epsilon_{\perp}}-1}{\sqrt{\epsilon_{||}\epsilon_{\perp}}+1},\quad t_{\mathrm{c}}=\frac{1}{\sqrt{\epsilon_{||}\epsilon_{\perp}}+1}. \quad (20)$$

To find the electron energy loss, we numerically integrate $P_{\mathrm{bulk}}$, $P_{\mathrm{guid}}$ and $P_{\mathrm{begr}}$ according to Eq. (1) of the main text.
By setting $\epsilon_{||}=\epsilon_{\perp}=\epsilon$ one recovers EEL in isotropic material.

In Supplementary Figure 2 we show the comparison between $P_{\mathrm{bulk}}$ (Supplementary Figure 2a and e), $P_{\mathrm{guid}}$ (Supplementary Figure 2b and f), and the $P_{\mathrm{begr}}$ (Supplementary Figure 2c and g) for anisotropic and isotropic cases. The corresponding energy loss spectra after integration of $P(q,\omega)$ are shown in Supplementary Figure 2d and h, respectively. Note that in the isotropic case, $\Gamma_{\mathrm{guid}}(\omega)$ is attributed to the excitation of the surface phonon polariton (SPP) (as can be seen from the excellent match of SPP dispersion and the maximum of $P_{\mathrm{guid}}$ in Supplementary Figure 2f, for example). Nevertheless, the appearance of the main peak (marked by the dashed purple arrow in Supplementary Figure 2h) is very similar to that in anisotropic materials. Particularly, the peak position is also determined by the intersection of the electron line (purple line in Supplementary Figure 2f, inset) and the dispersion of the

(dominant) guided mode – SPP in this case (black dashed curve in Supplementary Figure 2f,g). Such universality of the peak formation mechanism (related to the guided wave excitation), as well as the appearance of the surface phonon peak at SO energy, will is explained in the next section.

**Supplementary Note 3: EEL due to guided wave excitation**

To explicitly relate $P_{\mathrm{guid}}(q,\omega)$ to the guided modes in the sample and find the peak position in the relevant energy loss spectrum $\Gamma_{\mathrm{guid}}(\omega)$ , we assume that the sample is thin, thus $\omega d/v \approx 0$. It is an excellent approximation at mid-IR frequencies, since the transmission STEM applies to samples with thickness $\lesssim 100$ nm and utilizes electron with speed $v\sim c/2$. In addition, we neglect $\omega^2/v^2$ compared to $\sqrt{\epsilon_\perp/\epsilon_{||}}\,q^2$, which essentially extends the validity of the quasistatic approximation to momenta near the light cone ($q\sim\omega/c$) due to large values attained by $|\epsilon_\perp|$ in the low-frequency part of the Reststrahlen band (see Fig. 2 of the main text). Upon substitution of coefficients $A_3$ and $B_1$ [Eqs. (17-18)] into Eq. (11) and application of these approximations we obtain:

$$P_{\mathrm{guid}}(q,\omega) \approx \frac{e}{\pi^2\hbar v}\frac{q^2v^2}{(q^2v^2+\omega^2)^2}\,\mathrm{Im}\left[\frac{\sqrt{\epsilon_{||}\epsilon_\perp}\left(1-r_{\mathrm{p}}\mathrm{e}^{-2\sqrt{\frac{\epsilon_\perp}{\epsilon_{||}}}qd}-2t_c\mathrm{e}^{-\sqrt{\frac{\epsilon_\perp}{\epsilon_{||}}}qd}\right)}{\left(1+\sqrt{\epsilon_{||}\epsilon_\perp}\right)\left(1-r_{\mathrm{p}}^2\mathrm{e}^{-2\sqrt{\frac{\epsilon_\perp}{\epsilon_{||}}}qd}\right)}\right]. \quad (21)$$

By further noticing that for large $|\epsilon_\perp|$, we have $r_{\mathrm{p}} \approx \sqrt{\epsilon_{||}\epsilon_\perp}/(1+\sqrt{\epsilon_{||}\epsilon_\perp}) \sim 1$ and $t_{\mathrm{c}}\sim 0$, which yields:

$$P_{\mathrm{guid}}(q,\omega) \approx \frac{e}{\pi^2\hbar v}\frac{q^2v^2}{(q^2v^2+\omega^2)^2}\,\mathrm{Im}\left[\frac{r_{\mathrm{p}}\left(1-\mathrm{e}^{-2\sqrt{\frac{\epsilon_\perp}{\epsilon_{||}}}qd}\right)}{1-r_{\mathrm{p}}^2\mathrm{e}^{-2\sqrt{\frac{\epsilon_\perp}{\epsilon_{||}}}qd}}\right] \quad (22)$$

where we can immediately recognize the (quasistatic) reflection coefficient from an anisotropic slab[5], $R_{\mathrm{p}}$:

$$R_{\mathrm{p}}(q,\omega) = \frac{r_{\mathrm{p}}\left(1-e^{-2\sqrt{\frac{\epsilon_\perp}{\epsilon_{||}}}qd}\right)}{\left(1-r_{\mathrm{p}}^2e^{-2\sqrt{\frac{\epsilon_\perp}{\epsilon_{||}}}qd}\right)}. \quad (23)$$

The guided modes are defined as the divergences (poles) of $R_\mathrm{p}(q,\omega)$, which for real frequencies occur at complex-valued momenta $q$ that turn the denominator of $R_\mathrm{p}$ into zero. The connection between these momenta and the frequency (mode energy), $q = q_\mathrm{M}(\omega)$, represents the dispersion of the guided mode (M is the mode index). For real momenta $q = \mathrm{Re}(q_\mathrm{M})$, $R_\mathrm{p}$ does not diverge, but rather acquires a large imaginary part. Therefore according to Eq. (22), the dominant contribution to $P_\mathrm{guid}$ comes from the points in $(q,\omega)$ space that lie near the dispersion of the guided modes. This establishes the formal connection between these modes and $P_\mathrm{guid}$ (and thus $\Gamma_\mathrm{guid}$).

One can employ the pole approximation[6] to formally evaluate the integral in Eq. (1) of the main text and find $\Gamma_\mathrm{guid}(\omega)$:

$$\Gamma_\mathrm{guid}(\omega) = \int dq P_\mathrm{guid}(q,\omega) = 2\pi \mathrm{i} \sum\nolimits_\mathrm{M} \mathrm{Res}\left[P_\mathrm{guid}(q,\omega)\right]_{q=q_M(\omega)}. \tag{24}$$

For thin samples and relatively low momenta, we have $qd \ll 1$. Therefore, the leading term in Taylor expansion (in $\xi = qd$) of the numerator in $R_\mathrm{p}$ [Eq. (23)] is $2qd\sqrt{\frac{\epsilon_\perp}{\epsilon_{||}}} r_\mathrm{p} \propto q$. It is thus useful to pull the factor $q$ out of $R_\mathrm{p}$ and define the function $F(q,\omega)$ as:

$$F(q,\omega) = \frac{e}{\pi^2 \hbar v} \frac{q^3 v^2}{(q^2 v^2 + \omega^2)^2}, \tag{25}$$

yielding $P_\mathrm{guid}(q,\omega)$ as written in Eq. (3) of the main text. With such definition, $\mathrm{Res}\left[P_\mathrm{guid}(q_M(\omega),\omega)\right]$ becomes directly proportional to $F(q_M(\omega),\omega)$ (for each fixed frequency $\omega$). The spectral shape of $\Gamma_\mathrm{guid}(\omega)$ is thus largely determined by $F$. Particularly, the spectral peak position, $\omega_\mathrm{g}$, in $\Gamma_\mathrm{guid}$ corresponds to the maximum of $F(q_M(\omega),\omega)$. Since $\max F(q,\omega)$ occurs when $q = q_\mathrm{max}(\omega) = \sqrt{3}\omega/v$, $\omega_\mathrm{g}$ can be can be obtained from the equation:

$$\frac{\sqrt{3}\omega_\mathrm{g}}{v} = q_\mathrm{M}(\omega_\mathrm{g}). \tag{26}$$

The solution to this equation can be found geometrically as the intersection of the line defined by $q = q_\mathrm{max}$ with the mode dispersion $q_\mathrm{M}(\omega)$, as described in the main text.

For hyperbolic materials, such as h-BN in the Reststrahlen bands, the guided modes are hyperbolic polaritons (HPs). Within quasistatic approximation their dispersion in the h-BN slab can be written as[5]:

$$q_\mathrm{M}(\omega) = \mathrm{i}\frac{\sqrt{\epsilon_{||}}}{d\sqrt{\epsilon_\perp}}\left(2\arctan\frac{\mathrm{i}\sqrt{\epsilon_\perp}}{\epsilon_\perp\sqrt{\epsilon_{||}}} + \pi \mathrm{M}\right) \tag{27}$$

where M is the mode index. For illustration we perform a calculation for 30 nm thick h-BN slab. In Supplementary Figure 3a we see that the dominant contribution to $\mathrm{Im}\left[q^{-1}R_\mathrm{p}(q,\omega)\right]$ comes from the M0-HPhP mode. Higher order modes (shown in the inset using saturated color scale), while presents, provide insignificant contribution at low momenta (where $F$ exhibits its maximum, see Supplementary Figure 3b) and can be neglected. The major peak in $\Gamma_\mathrm{guid}(\omega)$ is thus determined by the intersection of the "electron" line with the M0-HPhP mode dispersion as depicted in Supplementary Figure 3c.

Note that in thin films, $\Gamma_{\text{guid}}$ is determined by the polariton dispersion at relatively low momenta ($q \sim \omega/v$ near the light cone), since the contribution from large momenta is suppressed by $1/q$ decay of $F(q,\omega)$. This however, is only true when $R_{\text{p}}(q,\omega)$ does not exhibit strong resonances in the quasistatic limit $q \to \infty$. In this limit, $R_{\text{p}}$ reduces to the quasistatic reflection coefficient from a semiinfinite slab $r_{\text{p}}$ defined in Eq. (20). For h-BN and all other vdW materials in hyperbolic regime ($\epsilon_{||}\epsilon_{\perp} < 0$, meaning the real parts), the real part of the denominator in $r_{\text{p}}$ never approaches zero as a function of frequency, resulting in the absence of resonant behavior (see Supplementary Figure 4a). This is in sharp contrast to isotropic media, where $\text{Re}[\epsilon(\omega) - 1]$ can reach zero, resulting in the strong resonance in $r_{\text{p}}$. This resonance is called the surface resonance and the frequency at which it occurs marks the SO energy (see Supplementary Figure 4b). This resonance could provide a large contribution to the integral in Eq. (1) of the main text, resulting in an additional peak in $\Gamma(\omega)$ at SO energy (see Supplementary Figure 2h).

Note, that the lack of quasistatic resonance in the reflection coefficient of hyperbolic materials also explains the absence of the SO resonance peak in their nearfield spectra (as obtained by scattering-type scanning nearfield optical microscopy for example)[5,7], in contrast to nearfield spectra of isotropic materials where SO resonance provides the dominant contribution[8,9].

**Supplementary Note 4: Bulk loss peak and the begrenzungseffekt**

The bulk loss peak at LO energy in $\Gamma_{\text{bulk}}(\omega)$ (blue in Supplementary Figure 2d,h) originates from the asymptotic behavior of $P_{\text{bulk}}$ at large momenta:

$$P_{\text{bulk}}(q \gg \omega/v, \omega) \propto -\text{Im}\left(\frac{1}{\epsilon_{\perp}}\right) \tag{28}$$

which provides a strong contribution to bulk loss at LO energy (see Supplementary Figure 2a) where $\text{Re}(\epsilon_{\perp}) = 0$. This result is identical to that obtained for isotropic systems (see Supplementary Figure 2e), such as metals or polar crystals[3]. However, in contrast to isotropic materials, we also see a contribution to $P_{\text{bulk}}$ at energies throughout the whole Reststrahlen band coming from low momenta ($q < 10^5 \text{cm}^{-1}$ in Supplementary Figure 2a). This can be explained by the presence of a pole on the real q-axis in $P_{\text{bulk}}$ for $\epsilon_{\perp}\epsilon_{||} < 0$ [meaning real parts, see Eq. (28)]. This pole can be associated with an excitation of the volume polariton mode in unbounded (bulk) hyperbolic media. Such volume polaritons are not supported in isotropic media (no real poles for $\epsilon_{\perp}\epsilon_{||} > 0$) and therefore $P_{\text{bulk}}$ does not show the corresponding loss (Supplementary Figure 2e).

The excitation of the bulk polariton results in a non-Lorentzian shape of the bulk loss peak, $\Gamma_{\text{bulk}}(\omega)$ (blue curve in Supplementary Figure 2d). To exemplify this behavior, we carry out the momentum integral in $\Gamma_{\text{bulk}}(\omega)$ analytically and obtain:

$$\Gamma_{\text{bulk}}(\omega) = -\frac{de^2}{(2\pi)^2 \hbar v^2 \epsilon_0} \text{Im}\left[\frac{1}{\epsilon_{\perp}} \ln\left(\frac{q_c^2 v^2 \epsilon_{\perp}}{\omega^2 \epsilon_{||}} + 1\right)\right]. \tag{29}$$

We can see that the asymmetry of the bulk loss in anisotropic materials stems from the logarithmic term in the right hand side of Eq. (29) and is more pronounced for small cutoff apertures.

Similarly to the bulk loss, we can obtain a crude approximation for $P_{\text{begr}}(q,\omega)$ by assuming that $q \to \infty$, resulting in

$$P_{\mathrm{begr}}\left(q \gg \frac{\omega}{v}, \omega\right) \approx -P_{\mathrm{bulk}}(q \gg \frac{\omega}{v}, \omega) \propto \mathrm{Im}\left(\frac{1}{\epsilon_{\perp}}\right), \tag{30}$$

which explains the near complete cancelation of the bulk loss in $\Gamma(\omega)$ (depicted in Supplementary Figure 2d) in anisotropic (as well as isotropic materials) materials.

### Supplementary Note 5: Retarded solution

To obtain the retarded solution for $\mathbf{E}_{\mathrm{ind}}$, we have to solve the electric field using full set of the Maxwell's equations. By eliminating magnetic field, we obtain the wave equation for the total electric field, $\mathbf{E}(\mathbf{r}, t)$, in the anisotropic system:

$$\boldsymbol{\nabla}(\boldsymbol{\nabla} \cdot \mathbf{E}) - \nabla^2 \mathbf{E} = -\mu_0 \left(\frac{\partial \mathbf{J}_{\mathrm{f}}}{\partial t} + \epsilon_0 \frac{\partial^2 (\hat{\epsilon}\mathbf{E})}{\partial t^2}\right), \tag{31}$$

where $\mathbf{J}_{\mathrm{f}} = \mathbf{v}\rho_{\mathrm{f}} = \hat{z}v(-e\delta[\mathbf{r} - \mathbf{r}_{\mathrm{t}}(t)])$ is the current that corresponds to the electron moving along the $z$ axis. Similarly to the non-retarded case, we utilize the Fourier transform and solve for the electric field separately outside and inside the anisotropic slab. Components of the field (parallel to the $z$ axis and the radial component) read:

$$E_{z,1} = \frac{iq}{\alpha_0} B_{1,\mathrm{ret}} \exp(-\alpha_0 z) + \left(1 - \frac{q^2}{\frac{\omega^2}{v^2} + \alpha_0^2}\right) \frac{\mathrm{i}e}{\epsilon_0 \omega} \exp\left(\frac{\mathrm{i}\omega z}{v}\right), \tag{32}$$

$$E_{R,1} = B_{1,\mathrm{ret}} \exp(-\alpha_0 z) + \frac{\mathrm{i}qe}{\epsilon_0 v \left(\frac{\omega^2}{v^2} + \alpha_0^2\right)} \exp\left(\frac{\mathrm{i}\omega z}{v}\right), \tag{33}$$

$$E_{z,2} = -\frac{\mathrm{i}q}{\alpha} \frac{\epsilon_{\perp}}{\epsilon_{||}} \left(A_{2,\mathrm{ret}} \exp(\alpha z) - B_{2,\mathrm{ret}} \exp(-\alpha z)\right) + \left(1 - \frac{\epsilon_{\perp}}{\epsilon_{||}} \frac{q^2}{\frac{\omega^2}{v^2} + \alpha_0^2}\right) \frac{\mathrm{i}e}{\epsilon_0 \omega \epsilon_{||}} \exp\left(\frac{\mathrm{i}\omega z}{v}\right), \tag{34}$$

$$E_{R,2} = A_{2,\mathrm{ret}} \exp(\alpha z) + B_{2,\mathrm{ret}} \exp(-\alpha z) + \frac{\mathrm{i}qe}{\epsilon_0 v \epsilon_{||} \left(\frac{\omega^2}{v^2} + \alpha^2\right)} \exp\left(\frac{\mathrm{i}\omega z}{v}\right), \tag{35}$$

$$E_{z,3} = -\frac{\mathrm{i}q}{\alpha_0} A_{3,\mathrm{ret}} \exp(\alpha_0 z) + \left(1 - \frac{q^2}{\frac{\omega^2}{v^2} + \alpha_0^2}\right) \frac{\mathrm{i}e}{\epsilon_0 \omega} \exp\left(\frac{\mathrm{i}\omega z}{v}\right), \tag{36}$$

$$E_{R,3} = A_{3,\mathrm{ret}} \exp(\alpha_0 z) + \frac{\mathrm{i}qe}{\epsilon_0 v \left(\frac{\omega^2}{v^2} + \alpha_0^2\right)} \exp\left(\frac{\mathrm{i}\omega z}{v}\right), \tag{37}$$

where we denote

$$\alpha^2 = \frac{\epsilon_{\perp}}{\epsilon_{||}} q^2 - \frac{\omega^2}{c^2} \epsilon_{\perp}, \qquad \alpha_0^2 = q^2 - \frac{\omega^2}{c^2}. \tag{38}$$

The retarded coefficients are again found from the boundary conditions, now for the field components. We require continuity of the radial component of the electric field and the normal component of the dielectric displacement at the interfaces:

$$E_{R,1}\big|_{z=\frac{d}{2}} = E_{R,2}\big|_{z=\frac{d}{2}}, \quad E_{R,2}\big|_{z=-\frac{d}{2}} = E_{R,3}\big|_{z=-\frac{d}{2}}, \tag{39}$$

$$E_{z,1}\big|_{z=\frac{d}{2}} = \epsilon_{||}E_{z,2}\big|_{z=\frac{d}{2}}, \quad \epsilon_{||}E_{z,2}\big|_{z=-\frac{d}{2}} = E_{z,3}\big|_{z=-\frac{d}{2}}. \tag{40}$$

The final expressions for the retarded probabilities also split into three contributions:

$$P_{\text{guid,ret}}(q,\omega) = \frac{eq}{2\pi^2\hbar\omega}\text{Re}\left[\int_{-\infty}^{-d/2}\text{d}z\, E_{z,1}(q,z,\omega)\exp\left(\frac{-\text{i}\omega z}{v}\right) + \int_{d/2}^{\infty}\text{d}z\, E_{z,3}(q,z,\omega)\exp\left(\frac{-\text{i}\omega z}{v}\right)\right] = \frac{eq}{2\pi^2\hbar\omega}\text{Im}\left[\frac{qv}{\alpha_0}\left(A_{3,\text{ret}}\frac{\exp[-(\alpha_0-\text{i}\omega/v)d/2]}{\alpha_0 v-\text{i}\omega} - B_{1,\text{ret}}\frac{\exp[-(-\alpha_0+\text{i}\omega/v)d/2]}{\alpha_0 v+\text{i}\omega}\right)\right], \tag{41}$$

$$P_{\text{in,ret}}(q,\omega) = \frac{eq}{2\pi^2\hbar\omega}\text{Re}\left[\int_{-d/2}^{d/2}\text{d}z\, E_{z,2}(q,z,\omega)\exp\left(\frac{-\text{i}\omega z}{v}\right)\right] = P_{\text{begr,ret}}(q,\omega) + P_{\text{bulk,ret}}(q,\omega), \tag{42}$$

where

$$P_{\text{begr,ret}}(q,\omega) = \frac{eq}{2\pi^2\hbar\omega}\,\text{Im}\left[\frac{2vq}{\alpha}\frac{\epsilon_\perp}{\epsilon_{||}}\left(\frac{A_{2,\text{ret}}\sinh\left(\frac{\alpha d}{2}-\frac{\text{i}d\omega}{2v}\right)}{\alpha v-\text{i}\omega} - \frac{B_{2,\text{ret}}\sinh\left(\frac{\alpha d}{2}+\frac{\text{i}d\omega}{2v}\right)}{\alpha v+\text{i}\omega}\right)\right], \tag{43}$$

$$P_{\text{bulk,ret}}(q,\omega) = \frac{eq}{2\pi^2\hbar\omega}\,\frac{ed}{\epsilon_0\omega}\text{Im}\left[\left(1-\frac{q^2}{\frac{\omega^2}{v^2}+\alpha_0^2}\right) - \frac{1}{\epsilon_{||}}\left(1-\frac{\epsilon_\perp}{\epsilon_{||}}\frac{q^2}{\frac{\omega^2}{v^2}+\alpha^2}\right)\right]. \tag{44}$$

The retarded coefficients $A_{k,\text{ret}}$ and $B_{k,\text{ret}}$ are obtained from Eqs. (39-40). In Supplementary Figure 5 we show the influence of retardation both in anisotropic and isotropic case. We see that the retarded spectra are slightly redshifted, as expected, and also exhibit a feature below TO energy, which is related to Čerenkov radiation[2].

**Supplementary Note 6: Influence of the substrate**

To evaluate the influence of the substrate on the EEL spectra of h-BN, we repeated the non-retarded calculation described in the Supplementary Note 2 for a two-layer system, consisting of an isotropic substrate and an anisotropic layer. In Supplementary Figure 6 we compare spectra with and without the substrate. We considered a 30 nm thick layer and a 60 keV electron beam. The substrate is a 15 nm membrane characterized by a dielectric constant $\epsilon_{\text{sub}} = 4$, which is the permittivity of silicon nitride.[10] We see that including the substrate causes a small red-shift $\sim 0.5$ meV of the main energy loss peak due HPhP excitation and a slight intensity reduction of the bulk loss peak at LO.

**Supplementary References**


1. Ritchie, R. H. Plasma Losses by Fast Electrons in Thin Films. *Phys. Rev.* **106,** 874–881 (1957).
2. Lucas, A. . & Kartheuser, E. Energy-Loss Spectrum of Fast Electrons in a Dielectric Slab. I.Nonretarded Losses and Cherenkov Bulk Loss. *Phys. Rev. B* **1,** 3588–3598 (1970).
3. García de Abajo, F. J. Optical excitations in electron microscopy. *Rev. Mod. Phys.* **82,** 209–275 (2010).
4. Jackson, J. D. *Classical Electrodynamics*. (Wiley, 1999).
5. Dai, S. *et al.* Tunable Phonon Polaritons in Atomically Thin van der Waals Crystals of Boron Nitride.

*Science* **343,** 1125–1129 (2014).
6. Felsen, L. B. & Marcuvitz, N. *Radiation and Scattering of Waves*. (Wiley-Interscience, 2003).
7. Shi, Z. *et al.* Amplitude- and Phase-Resolved Nanospectral Imaging of Phonon Polaritons in Hexagonal Boron Nitride. *ACS Photonics* **2,** 790–796 (2015).
8. Hillenbrand, R., Taubner, T. & Keilmann, F. Phonon-enhanced light–matter interaction at the nanometre scale. *Nature* **418,** 159–162 (2002).
9. Zhang, L. M. *et al.* Near-field spectroscopy of silicon dioxide thin films. *Phys. Rev. B* **85,** 75419 (2012).
10. Kischkat, J. *et al.* Mid-infrared optical properties of thin films of aluminum oxide, titanium dioxide, silicon dioxide, aluminum nitride, and silicon nitride. *Appl. Opt.* **51,** 6789–6798 (2012).
